# Water Masses of the Arctic from 40 Years of Hydrographic Observations

**DOI:** 10.1038/s41597-026-06749-8

**Published:** 2026-02-14

**Authors:** Kate Oglethorpe, Joshua Lanham, Rafael S. Reiss, Emma J. D. Boland, Alberto C. Naveira Garabato, Colm-Cille P. Caulfield, Ali Mashayek

**Affiliations:** 1 University of Cambridge, Cambridge, UK; 2 British Antarctic Survey, Cambridge, UK; 3 University of Southampton, Southampton, UK; 4 National Oceanographic Centre, Southampton, UK

**Keywords:** Physical oceanography, Cryospheric science, Physical oceanography, Climate and Earth system modelling

## Abstract

The Arctic Ocean has been changing rapidly in a warming climate. To monitor these changes, it is useful to classify the Arctic Ocean into water masses-bodies of water with similar origin and physical and biogeochemical properties. However, there are significant barriers to Arctic water mass classification: observations of seawater properties are sparse, and traditional classification relies on extensive knowledge of water mass characteristics and circulation. To address these challenges, we compile existing hydrographic observations of the upper 1000 m of the Arctic Ocean and classify these observations into water masses. We present the classification tool and accompanying dataset, Water Masses of the Arctic (WMA), to support basin-wide investigations of Arctic Ocean circulation, its variability, drivers and impacts on wider Arctic climate. Our dataset reproduces key spatial and temporal features of Arctic water masses, including Atlantic and Pacific Water pathways. The WMA dataset will improve understanding of Arctic Ocean dynamics and provide an accessible framework for assessing the accuracy of the representation of the Arctic Ocean in Earth System Models.

## Background & Summary

The Arctic has warmed rapidly due to anthropogenic activity^1^, with significant regional and global implications^2^. Since 1979, the Arctic has lost nearly half of its sea ice volume^3^, leading to Arctic warming at over twice the global rate^1^, widespread habitat loss for Arctic marine species^4^ and a growing interest in the use of Arctic waters for shipping, fishing, extraction, and other commercial activities^5^. Beyond its regional impacts, Arctic change is expected to modify the global climate. The discharge of freshwater from the Greenland Ice Sheet and resultant surface freshening of the northern North Atlantic is expected to weaken the formation of the dense headwaters of the Atlantic Meridional Overturning Circulation (AMOC)^6–8^. Substantial weakening of the AMOC would have far-reaching physical, ecological, and societal consequences^9,10^.

**Fig. 1:**
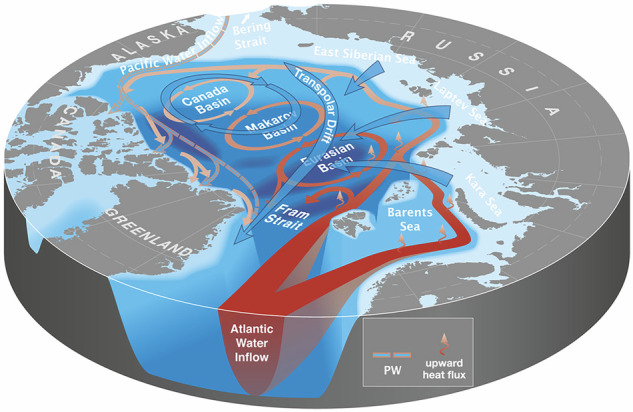
Schematic from Carmack *et al*.^9^ of the circulation of the surface water (blue), intermediate Pacific Water (pink/blue), and Atlantic Water (red) of the Arctic Ocean. ©American Meteorological Society. Used with permission.

The Carmack *et al*. schematic (Fig. 1) illustrates the essential Arctic Ocean water mass distribution. Arctic surface water (ASW) is exceptionally cool and fresh due to intense atmospheric cooling and large river input. The low temperatures of the Arctic mean that salinity rather than temperature dominates seawater density^11^. Thus, the warm and saltier Atlantic Water (AW) flowing north through the Fram Strait and Barents Sea Opening subducts under the fresher ASW^12^. This AW supplies most of the oceanic heat entering the Arctic Ocean^13^. Pacific Water (PW) flowing through the Bering Strait also subducts and contributes to the warm subsurface layer in the Canada Basin. A strong halocline separates the warm AW and PW and cold ASW across most of the Arctic Ocean^12^.

Arctic water masses are changing rapidly through two primary mechanisms. First, through modified atmosphere-ocean and ice-ocean fluxes via prolonged open-ocean and marginal-ice-zone conditions, leading to: i) enhanced solar warming of surface and halocline waters, so far observed in the Chukchi and Barents Seas^14^, ii) enhanced wind-driven vertical mixing and upward transport of AW heat, so far observed in the Eurasian and Makarov Basins,^15,16^, and iii) amplification of currents and eddy activity, with potential enhancement of vertical heat exchange^17,18^. Second, Arctic water masses are modified through changes in the properties and volume of inflowing waters. Inflowing AW has warmed and shoaled significantly over the twentieth century^19,20^, facilitating greater vertical heat transfer to the overlying ASW^19,20^. The Canada Basin is also affected by the increasing volume and heat content of PW and northward retreat of the cold halocline layer^21^. These climate-driven transformations directly impact Arctic sea ice and the formation rates of dense waters of the AMOC, yet their full implications are not yet fully understood^12^.

A water mass is a body of water formed by a single formation process, primarily surface forcing. These processes imprint physical and biogeochemical properties that are characteristic of the water mass. Physical properties such as absolute salinity (SA) and conservative temperature (CT) are quasi-conserved and subsequently modified only by mixing with other water masses^22^. In contrast, biogeochemical properties, including dissolved oxygen (DO), carbon and nutrients, are modified by both mixing and biological processes^23^. These physical properties govern the Arctic Ocean stratification and vertical heat and freshwater exchange that controls sea ice dynamics and regional climate feedbacks^12^. These water mass biogeochemical properties impact Arctic ecosystems and efficiency of the Arctic Ocean biogeochemical carbon pump^24^. Therefore, the classification of observations into water masses is fundamental to monitor and understand these climate-driven Arctic water mass transformations.

However, our ability to quantify Arctic water masses from observations is poor, hindering our ability to monitor Arctic change. The observations required to define the physical and biogeochemical properties of these water masses^22^ are sparsely and heterogeneously sampled in space and time in the Arctic Ocean^12^. This limited coverage is largely due to the region’s extreme environmental conditions which make sustained observations logistically challenging and seasonally biased. Moreover, the most common water mass classification method, which estimates relative water mass fractions via a least squares analysis, demands an extensive knowledge of water mass characteristics, circulation, and mixing^25,26^. Specifically, the least squares-based Optimum Multiparameter (OMP) analysis requires good knowledge of the properties of the unmixed water masses. To date, OMP analysis has only been applied in regional contexts in the Arctic: for example, to intermediate waters in the Nordic Seas using ship data^27^, intermediate and deep waters in the central Arctic using a World Ocean climatology^28^, water masses of the East Greenland Current from a single cruise^29^, and water masses in the Beaufort Sea from a single cruise^30^. Extending such approaches to the entire Arctic remains difficult due to the scarcity of basin-wide observations.

At present, no publicly available, observation-based, basin-wide dataset of Arctic Ocean water masses exists. To address this gap, we compile a variety of existing hydrographic observations from the upper 1000 m of the Arctic Ocean, classify them into water masses, and share both the resulting dataset and the classification tool. This tool-previously developed by Lanham *et al*.^31,32^-semi-automates traditional water mass classification by first estimating relative water mass fractions using traditional OMP analysis of CT, SA, and DO observations. These estimates are then used to train a supervised machine learning (ML) model, which classifies the remaining CT and SA observations (where DO is unavailable) with inclusion of additional features: location, depth, and season. This hybrid approach extends the reach of classical OMP methods, enabling a basin-wide classification of Arctic Ocean water masses for the first time.

Our observational WMA dataset enhances our ability to characterise and monitor the changing dynamics of the Arctic Ocean. The accompanying classification tool also provides a framework for generating updated water mass estimates as new hydrographic observations become available. With appropriate adjustments, the tool can also be applied to Earth System Model (ESM) output, supporting the verification of climate simulations. Together, the dataset and tool provide a reproducible and adaptable resource for Arctic water mass analysis.

## Methods

### Arctic Observations

Publicly available observations of CT, SA, and DO concentration from the top 1000 m of the Arctic Ocean were compiled and used in this study. In-situ temperature and salinity are converted into CT and SA, respectively, using the thermodynamic equation of seawater using the routines of Jacket and McDougall^33^. The observation sources and spatial and temporal distributions are described in Table 1 and Fig. 1, respectively. A detailed breakdown of the distributions for each data source and the relative contributions of each data source is provided in Figures S1-4 of the Supplementary Information. The Python code for compilation is available through Zenodo: 10.5281/zenodo.17234454^34^. The final compiled observations are provided in the water mass dataset generated by this study, titled Water Masses of the Arctic (WMA), available through Figshare: 10.6084/m9.figshare.29646629^35^.

**Table 1:** Sources of Arctic Ocean observations of temperature, salinity, and dissolved oxygen compiled for this study.

Program	Date	Platform type	Instrument	Data Access	QC documentation
Ice-Tethered-Profilers (ITP)^72^	2004–2024	autonomous profiler	CTD sensor package	Level 3 data https://www2.whoi.edu/site/itp/data/	https://www2.whoi.edu/site/itp/data/data-products/
Argo^73^	2001–2024	autonomous profiler	CTD sensor package	https://dataselection.euro-argo.eu	https://www.argodatamgt.org/Documentation.html
Global Ocean Data Analysis Project (GLODAP)^36^	1980–2021	ship	CTD sensor package & chemical analysis of seawater samples	https://www.ncei.noaa.gov/data/oceans/ncei/ocads/data/0257247/	https://essd.copernicus.org/articles/14/5543/2022/
Unified Database for Arctic and Subarctic Hydrography (UDASH)^37^	1980–2015	various	various, mostly CTD sensor package & expendable bathythermographs	10.1594/PANGAEA.872931	–
MOSAiC expedition^74^	2019–2020	ship	CTD sensor package, chemical analysis of seawater samples	10.1594/PANGAEA.959964	–

The Unified Database for Arctic and Subarctic Hydrography includes some data originally from Ice-Tethered Profilers (ITP) and Argo, as well as ship-based data that may also appear in Global Ocean Data Analysis Project (GLODAP); any duplicates are removed from UDASH during our compilation to ensure each observation is represented only once. The Quality Control (QC) documentation for UDASH and MOSAiC are found in the data source URLs. The ITP product used here is Level 3 processed data.

All data used in this study are derived from publicly available, pre-processed hydrographic products, rather than raw sensor output. For each data source-Argo, Ice-Tethered Profiler (ITP), Global Ocean Data Analysis Project (GLODAP), MOSAiC, and UDASH-the products have undergone extensive quality control (QC) procedures (Table 2; column 5), including calibration, sensor correction, and removal or flagging of obviously erroneous data. The ITP product used here is to Level 3 processed data.

**Table 2:** SWTs for prominent water masses occupying the top 1000 m of the Arctic Ocean, used in this water mass classification.

Source Water Type (SWT)	CT (°C)	SA (g/kg)	DO (*μ*mol kg^−1^)	Associated Water Mass (literature-informed)	
				Qualitative Summary	Quantitative Criteria
Arctic Surface Water (ASW)	−1.45	27.00	390.3	Cold and fresh surface layer formed by atmospheric cooling, melting of sea ice, and freshening by river discharge and precipitation.	*σ*_0_ < 26.0 kgm^−3^^84^; T < 0°*C*^75,76^; surface to 25-50 m^45^; DO maximum^77^.
Summer Pacific Water (sPW)	6.00	31.20	314.0	Warm and fresh (relative to Atlantic) Pacific-origin water flowing north through the Bering Strait into the Arctic Ocean in summer.	S = 32-33 PSU^42^; T_*max*_ within S = 31-33 PSU^78^; T set in the Chukchi Sea^79^; S < 33.64^80^; S = 30.72-31.40 g/kg^81^.
Modified Summer Pacific Water (MsPW)	0.50	31.00	354.2	Cooler flavour of Pacific-origin water residing in the Beaufort Gyre.	T = −1-1°*C*^12^; 30-100 m and T > 0°*C*^82^; 50-100 m and T_*max*_ within depth range^45^; S < 33.64^80^.
Winter Pacific Water (wPW)	−1.50	33.20	280.8	Cold and fresh (relative to Atlantic) Pacific-origin water flowing north through the Bering Strait into the Arctic Ocean in winter.	*σ*_0_ < 27.4 kgm^−3^; S = 33.1 PSU^45^; S = 31.8-33.2 PSU and T = −1.8 to -0.2°*C*^82^; S < 33.64^80^; S = 33.01-33.41 g/kg^81^; DO minimum^77^; T set in the Chukchi Sea^79^.
Norwegian Current Water (NCW)	8.00	35.40	287.8	Warm and saline Atlantic-origin water flowing north through the Fram Strait or Barents Sea into the Arctic Ocean.	T = 7-9°*C* and S = 35.2 PSU^45^; T_*max*_ = 8°*C* and S = 34-35 PSU^84^.
Atlantic Water (AW)	0.00	35.05	298.0	Cooler flavour of Atlantic-origin water residing below the halocline across the Arctic Ocean.	T > 0°*C*^20^; S > 34.9 PSU; T = 0.4-3°*C* and 200-900 m^45^; *σ*_0_ = 27.1-30.28 kg m^−3^^84^; T = −1.8-2.5°*C*^82^; T > 0°*C* and S = 34.5-35.0 PSU^75^; T = 0-3°*C* and 150-500 m^12^; T ≥ 2°*C* and S ≥ 34.7 PSU^76^; T > 0°*C* and S = 34.8-35.0^83^; S > 33.64^80^; S = 34.98-34.99^81^.
Brine-enriched Water (BW)	−1.80	34.45	370.0	Cold and salty brine-enriched layer formed by surface cooling, freezing, and subsequent convective mixing.	T = T_*freezing*_^84^; T < −1.5 and S > 34.8 PSU^85^.

SWTs represent the unmixed CT, SA, and DO properties of water masses. SWT definitions are based on observed extremes in CT-SA-DO space and literature on Arctic Ocean water mass structure (columns 5 and 6). *σ*_0_ denotes potential density when the reference pressure is the surface (0 dbar). Note that water mass criteria vary substantially across studies due to differences in regional focus, data sources, and the inherently subjective nature of water mass definitions. Although fixed depth ranges for water masses are given here for context, we do not use them to define SWTs due to the interior dynamic environment.

The QC procedures vary across the data products, and the Unified Database for Arctic and Subarctic Hydrography (UDASH) product includes data from various archives with different types of QC procedures. Therefore, after merging UDASH with the other data sources, we implement our own QC protocol across the merged dataset. This involves removing salinity values greater than 38 g/kg and less than 10 g/kg and temperature values less than −2 °C and greater than 15 °C. These limits follow those applied in GLODAP’s QC procedures^36^ and detect single-point outliers that may not have been removed during prior processing. While UDASH includes its own flagging system, these quality tests can be overly sensitive, sometimes causing profiles to fail one (or more) checks. Applying our own QC ensures maximum usable data coverage and consistency across all datasets. Additional gradient-based outlier detection following past QC procedures for Arctic hydrographic datasets^37–39^ removed only a negligible fraction (0.66%) of data and did not affect the results, so it was not retained in our final QC procedure.

Our QC procedure also identifies and removes duplicate data in our merged dataset. This is necessary because UDASH incorporates some data originally from ITP and Argo, as well as ship-based data that may also appear in GLODAP. We used the Argo, ITP, and GLODAP datasets rather than their UDASH duplicates because DO measurements were not available in UDASH. There were no duplicate profiles between the MOSAiC and other datasets.

Our final stage of processing involves averaging all profiles into 10 m bins. This reduces spatial and temporal biases caused by uneven vertical sampling resolutions between data sources, particularly high-density ITP and Argo data (typical vertical spacing  ~25 cm) compared with ship-based GLODAP and UDASH data (vertical spacing ranging from centimetres to hundreds of metres). The vertical resolution of each data source prior to averaging is shown in Figure S3. Although these data biases do not affect our OMP analysis as each data point is fit to the SWTs independently, biases can affect the interpretation of results with ITP and Argo overwhelming the results. We did not apply additional temporal binning because it would reduce horizontal spatial coverage by discarding observations.

UDASH, ITP, and GLODAP data contribute primarily to the final compiled dataset. For temperature and salinity profiles, UDASH, ITP, GLODAP, Argo, and MOSAiC contribute 65%, 33%, 2%, 0.1%, and 0.02%, respectively (Figure S4a in Supplementary Information). For DO profiles, ITP, GLODAP, Argo, and MOSAiC contribute 78%, 21%, 0.3%, and 0.02%, respectively (Figure S4b in Supplementary Information). The spatial and temporal distributions of UDASH, ITP, and GLODAP data vary considerably (Figures S1 and S2 in Supplementary Information). UDASH provides strong coverage in the GINS (Greenland-Iceland-Norwegian Seas), Barents Seas, Beaufort Gyre, Canadian shelves, and Bering Strait, but has notable gaps in the East Siberian and Laptev Seas and central Arctic. ITP is concentrated in the central Arctic and Beaufort Gyre. GLODAP data are concentrated in the GIN Seas, Beaufort Gyre, and Canadian shelves. Regarding seasonal coverage, UDASH and GLODAP are summer-biased, while ITP profiles are more evenly distributed throughout the year. Regarding inter-annual availability, both UDASH and GLODAP provide data since the 1980s, whereas ITP data is only available since the early 2000s.

Although MOSAiC contributes only a small fraction of the compiled dataset, it is valuable in our analysis because it covers undersampled regions of the eastern central Arctic during winter. Its inclusion also illustrates the potential to expand the dataset by adding additional cruises from both recent years and earlier decades. We acknowledge that our current compilation is not exhaustive; rather, it represents a deliberate selection of publicly available datasets to demonstrate the methodology. To facilitate future extensions, we provide the steps required to incorporate additional observations and re-run the water mass classification in Code Availability.

### Water Mass Classification

This section presents the two steps taken to generate our dataset of relative water mass fractions for the top 1000 m of the Arctic Ocean. First, relative fractions of water masses are estimated via an OMP analysis of the relatively small subset of observations containing CT and SA as well as DO concentrations. These estimates are then used to train a supervised ML model, which classifies the remaining CT and SA observations where DO is unavailable. Details of the tests used to evaluate the robustness of these steps are provided in the Technical Validation section Table 1.

#### Traditional water mass calculation

The relative fractions of Arctic Ocean water masses (0-1) are estimated via a non-negative least squares analysis of the CT, SA, and DO observations. This method, known as OMP analysis was originally implemented by Karstensen and Tomczak^40^. For this step, any observations where DO is not measured are excluded. The Python package used to implement the analysis is found in Shrikumar *et al*.^23^.

**Fig. 2:**
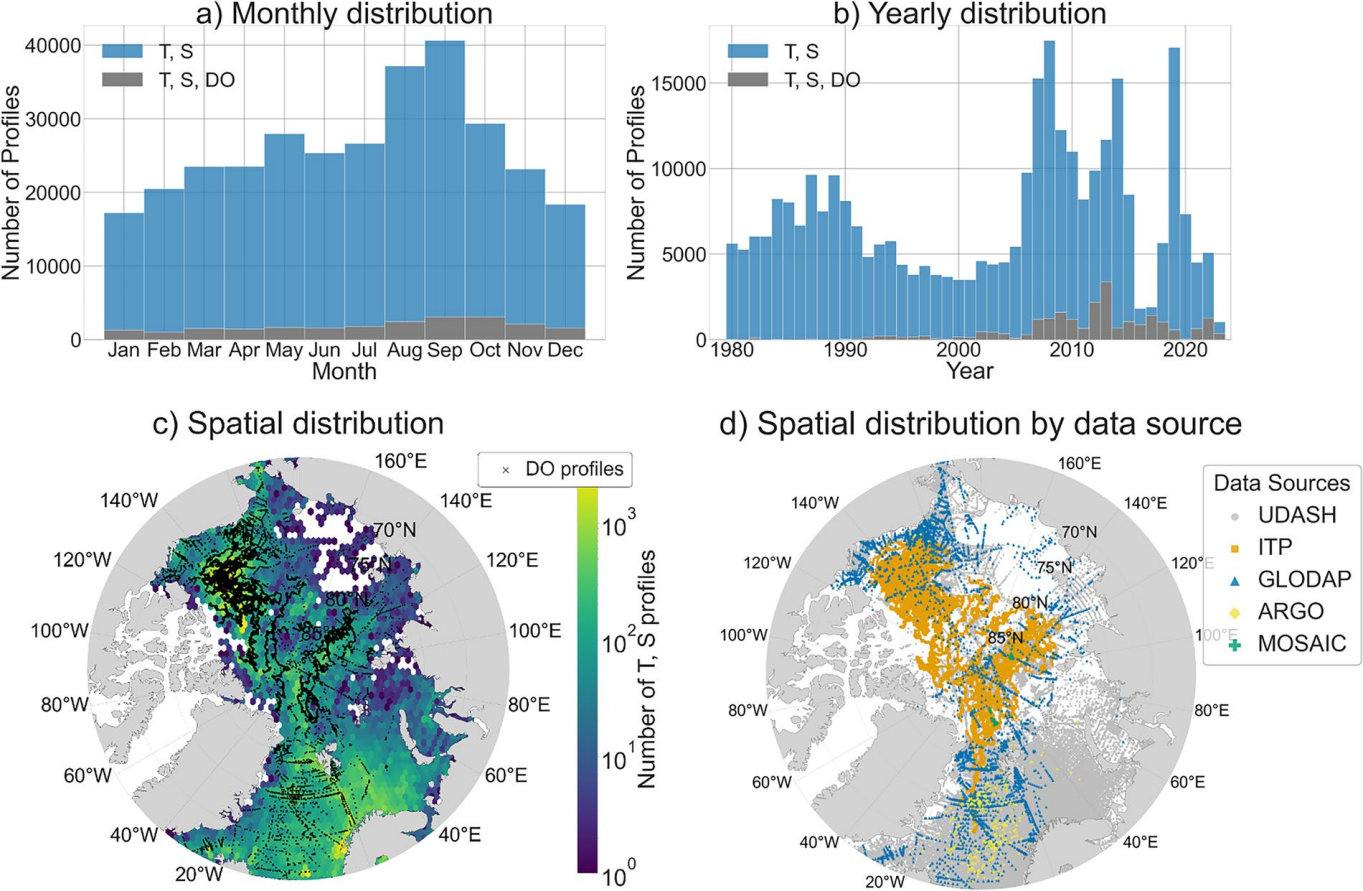
Temporal (top row) and spatial (bottom row) distributions of Arctic Ocean hydrographic observations used in this study: (**a**) monthly distribution, (**b**) yearly distribution, (**c**) spatial distributions coloured by number of T, S profiles per bin, and (**d**) spatial distribution coloured by data source. Grey bars (**a,b**) and black crosses (**c**) mark the times when and the sites where dissolved oxygen is measured, respectively. Hexagonal bins (**c**) cover approximately 3.6° in longitude and 0.25° in latitude. Data sources (**d**) are: Unified Database for Arctic and Subarctic Hydrography^37^ (UDASH; grey circle), Ice-Tethered Profilers^72^ (orange square), Global Ocean Data Analysis Project^36^ (blue triangle), Argo^73^ (yellow diamond), and MOSAiC^74^ (teal cross). UDASH includes some data originally from ITP and Argo, as well as ship-based data that may also appear in GLODAP; any duplicates are removed from UDASH during our compilation to ensure each observation is represented only once.

**Fig. 3:**
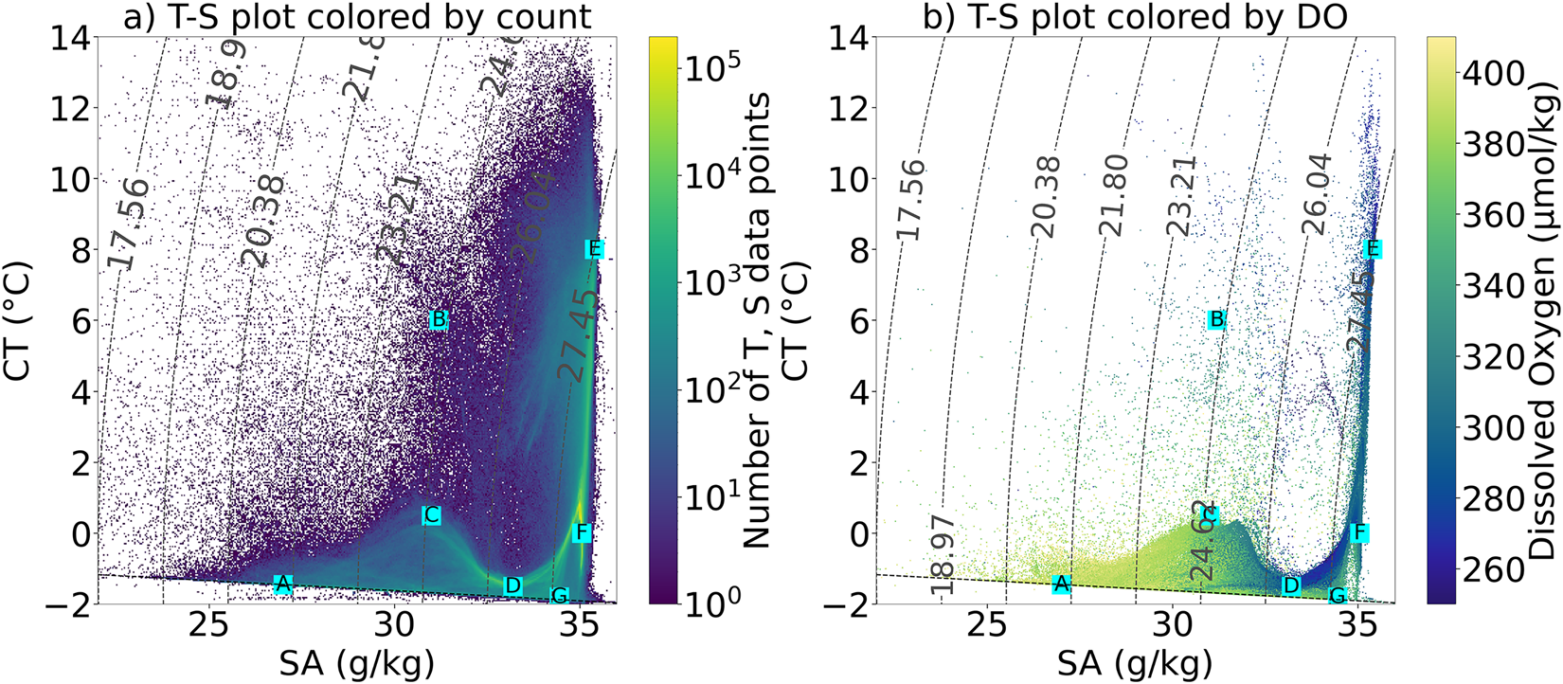
CT-SA diagrams of all Arctic observational data compiled for this study: (**a**) coloured by the number of observations on a logarithmic scale and (**b**) coloured by dissolved oxygen concentration (*μ*mol/kg), with data where dissolved oxygen is not measured being excluded. Cyan squares labeled A-G denote the Source Water Types (see Table 2), as follows: A - Arctic Surface Water (ASW), B - summer Pacific Water (sPW), C - Modified summer Pacific Water (MsPW), D - winter Pacific Water (sPW), E - Norwegian Current Water (NCW), F - Atlantic Water (AW), and G - Brine-enriched Water (BW). Dashed curved contours mark potential density referenced to a pressure of 0 dbar (surface) and the dashed horizontal line marks the freezing line.

The starting point of the classification method is the definition of properties of unmixed water masses, hereafter termed source water types (SWTs). SWTs are characterized by the extremes of CT, SA, and DO in CT-SA-DO space (Fig. 3). So, for each SWT, a single vector of CT, SA, and DO values is selected (columns 2-4 in Table 2) to represent the unmixed end member. Given notable spatial and temporal bias in observations (Fig. 2), arising from the Arctic’s challenging environment and the differing sampling strategies of individual data sources, literature on water mass properties and origins is also used to inform SWT definitions (Table 2). The quantitative criteria in the final column of Table 2 therefore represent literature-based ranges that informed, but did not prescribe, our choice of these single-point SWT values. The process is as follows:

1. Filter CT, SA, and DO data to retain only values within the geographic and/or property ranges of the water masses reported in the literature.
2. Within each filtered subset, manually identify CT and SA extremes in CT-SA space.
3. Assign the corresponding DO property value via nearest-neighbour analysis.

These steps were applied to the full dataset as well as separately to summer (July-September) and winter (January-March) subsets. From winter to summer, Norwegian Current Water (NCW) warms by  ~4 °C and Arctic Surface Water (ASW) freshens by  ~1.5 g/kg. summer Pacific Water (sPW) is present only in summer. We adopt winter SWT definitions for NCW and ASW, as they encompass the full annual property range: in summer, water mass fractions above the winter extremes would saturate to 1 regardless, while the winter definitions also remain valid for summer observations. Although sPW is absent in winter, it is retained in the year-round analysis to ensure correct classification of summer observations. Python scripts documenting the SWT selection are provided in Code Availability.

The fraction of each SWT at each observation point, *x*_*i*_, ranging 0 to 1, is estimated assuming the observed properties are a linear mixture of all the SWTs. The optimal combination of *x*_*i*_ is found in CT-SA-DO parameter space by minimizing residuals, R, in a non-negative least squares sense. This may be formulated using the following set of four linear mixing equations:

1 $$\mathop{\sum }\limits_{i=1}^{n}{x}_{i}\ast C{T}_{i}^{SWT}=C{T}^{obs}+{R}_{CT},$$

2 $$\mathop{\sum }\limits_{i=1}^{n}{x}_{i}\ast S{A}_{i}^{SWT}=S{A}^{obs}+{R}_{SA},$$

3 $$\mathop{\sum }\limits_{i=1}^{n}{x}_{i}\ast D{O}_{i}^{SWT}=D{O}^{obs}+{R}_{DO},$$

4 $$\mathop{\sum }\limits_{i=1}^{n}{x}_{i}=1+{R}_{{\rm{Mass}},}$$

where *n* is the number of SWTs, *CT*^*obs*^, *SA*^*obs*^, and *DO*^*obs*^ are the observed properties, $C{T}_{i}^{SWT}$, $S{A}_{i}^{SWT}$, and $D{O}_{i}^{SWT}$ are the *SWT*_*i*_ properties, *x*_*i*_ is the relative fractions of *SWT*_*i*_ and *R*_*SA*_, *R*_*CT*_, and *R*_*DO*_ are the residuals. The last row expresses the condition of mass conservation.

Each equation is weighted to account for confidence in the SWT and observed properties. Details of the weighting procedures are found in a Southern Ocean water mass classification Lanham *et al*.^41^.

These equations are then reformulated in terms of *R* (e.g., *R*_*SA*_) and solved using a least-squares optimisation to minimise *R*. Only solutions satisfying *x*_*i*_ ≥ 0 are accepted so that analysis is ’non-negative’. The Pyompa python package, used for this implementation, uses a hard mass constraint, thereby prioritising the return of a residual of 0 in the mass equation to comply with mass conservation. The optimisation is performed simultaneously for all SWTs, yielding a matrix of SWT (or water mass) fractions, *X* and matrix of residuals, *R*. For further details on constraints and optimisation, see Lanham *et al*.^41^ and Shrikumar *et al*.^23^. The residuals of the solution can be found in Text S1 and Figure S5 of the Supplementary Information.

We acknowledge that the assumption of conservative behaviour for DO, CT, and SA used in this analysis will not hold in some regions and time periods for several factors:

- DO is influenced by biological processes^22^ and typically has greater measurement uncertainties than CT and SA. To address this, we follow Lanham *et al*.^41^ by assigning lower weights to DO parameters compared to CT, SA, and mass parameters. As such, we reduce the influence of oxygen on the solution relative to conservative tracers. Sensitivity of the solution to these weightings is evaluated in the Technical Validation.
- CT and SA can be modified by air-sea buoyancy fluxes near surface and ice shelves or glaciers, thus, as in other OMP analyses, confidence in water mass fractions is lower at these sites. We choose to retain these sites in our dataset following^41^ and discuss the influence of buoyancy fluxes on water mass estimates in the Technical Validation section.
- SWT properties can be influenced by non-conservative buoyancy flux trends which are influenced by anthropogenic forcing. Sensitivity of OMP output to long-term modifications of SWTs is evaluated in the Technical Validation.

This OMP analysis is significantly limited by the scarcity of DO observations, with DO present at only 10.3% of ST and CA observations compiled for this study (Fig. 3). However, DO remains a critical parameter in the OMP analysis, as it differentiates between wPW and BW which exhibit similar in CT and SA characteristics yet differ notably in DO concentration. wPW is relatively deoxygenated due to respiratory activity and nutrient regeneration at its formation site in the Chukchi Sea^42,43^, whereas BW is relatively oxygenated due to surface ventilation. Excluding DO from the OMP analysis results in up to a 50% reduction in the estimated wPW relative fractions at 50-250 m in the Canada Basin despite substantial wPW presence expected for this region^12^ (Figure S6 in Supplementary Information). The scarcity of DO measurements motivates the development of methods that can estimate water mass fractions from more widely available data, such as CT, SA, and depth measured by autonomous instruments. In this study, we pursue a ML approach for this purpose, as described in the following section.

#### Machine learning water mass calculation

This section provides a detailed summary of the ML extension to traditional OMP analysis, previously implemented for the Atlantic and Southern Oceans by Lanham *et al*.^31^ and Lanham et al^32^, respectively.

Following Lanham *et al*.^31^, we train a Random Forest five member model ensemble on relative water mass fractions derived from traditional OMP analysis, and use it to predict fractions from CT and SA observations where DO measurements are unavailable. The model ensemble is trained using the scikit-learn Python package^44^. Additional contextual features-including longitude, latitude, depth, and month-are included to improve prediction accuracy. In this way, the Random Forest model learns the thermohaline and spatio-temporal ‘fingerprints’ of the OMP-derived fractions, enabling their application to a dataset without DO data. This increases the number of usable observations for our water mass classification by an order of magnitude, dramatically expanding the spatio-temporal coverage of the water mass classification. Month is included but not year, ensuring the classification tool remains applicable to climatological datasets based on multi-year averages.

To ensure the robustness of the model, we use randomised 5-fold cross-validation, which follows these steps:

1. Prepare the training dataset to include desired features: CT, SA, longitude (converted to sine and cosine to handle 0/360° continuity), latitude, depth, month, and relative water mass fractions estimated by OMP water mass classification.
2. Randomise data before splitting. Split data into 5 equal-sized subsets (folds).
3. Perform 5-fold cross-validation by iterating over each fold: Train a Random Forest Regressor^44^ on 4 folds (80% of full dataset) to predict relative fractions of water masses. Predict water mass fractions for the remaining validation fold (20% of full dataset), and compute coefficient of determination, *R*^2^, to assess prediction accuracy. Store the trained model and its predictions for ensemble averaging.
4. Apply the trained models: Combine the predictions from all 5 trained models by computing the ensemble mean. Finally, use the trained ensemble model to predict water mass fractions for T and S observations where DO is not measured. Where DO is measured, we use the original OMP output of water mass fractions.

Prediction of water mass fractions (Steps 1-4) is performed for the full merged dataset and then repeated using two alternative training datasets: OMP-derived water masses estimates defined using (i) post-2010 data and (ii) pre-2000 data. This comparison allows us to assess how long-term changes in SWT properties influence model performance and predictions, as described in the Technical Validation.

Uncertainty in the ensemble mean relative water mass fractions is estimated using normalised variance across predictions from five independently trained Random Forest models generated by the 5-fold cross-validation procedure, along with spatial and temporal exclusion studies. These tests and their results are detailed in the Technical Validation.

## Data Records

The water mass dataset generated by this study, titled Water Masses of the Arctic (WMA), is available through Figshare: 10.6084/m9.figshare.29646629^35^. The dataset is provided in a Comma Separated Values (CSV) file format and contains all necessary information required to work with the data. A summary of the dataset can be found in Table 3.

**Table 3:** Variables provided in the dataset of Arctic Ocean water mass fractions.

Variable	Units	Description
Source	—	Source of publicly available T, S, and DO data
Nprof	—	Profile number
Longitude, Latitude	°E, °N	Geographic coordinates
Depth	m	Depth, positive downward
Datetime	UTC	Format: YYYY-MM-DD
CT	°C	Conservative temperature (TEOS-10)^86^
SA	g/kg	Absolute salinity (TEOS-10)^86^
DO	*μ*mol/kg	Dissolved oxygen
NCW	Fraction (0–1)	Norwegian Coastal Water (warm AW flavour)
AW	Fraction (0–1)	Atlantic Water (cool AW flavour)
ASW	Fraction (0–1)	Arctic Surface Water
BW	Fraction (0–1)	Brine-enriched Water
MsPW	Fraction (0–1)	Modified summer Pacific Water (cool sPW flavour)
sPW	Fraction (0–1)	Summer Pacific Water (warm sPW flavour)
wPW	Fraction (0–1)	Winter Pacific Water

The dataset is available through Figshare: 10.6084/m9.figshare.29646629^35^.

## Technical Validation

This section assesses the quality and robustness of our dataset of relative fractions of Arctic Ocean water masses through several approaches: 1) comparison of spatial and temporal distribution of our water mass fractions to prior knowledge of Arctic Ocean water mass pathways, 2) sensitivity tests to assess the subjective choices made in the OMP classification, specifically SWT definitions and weighting schemes, 3) ML model uncertainty, and 4) validation of the OMP analysis against a data-driven classification method.

### Comparison of water masses to prior knowledge

#### Spatial distribution

**Fig. 4:**
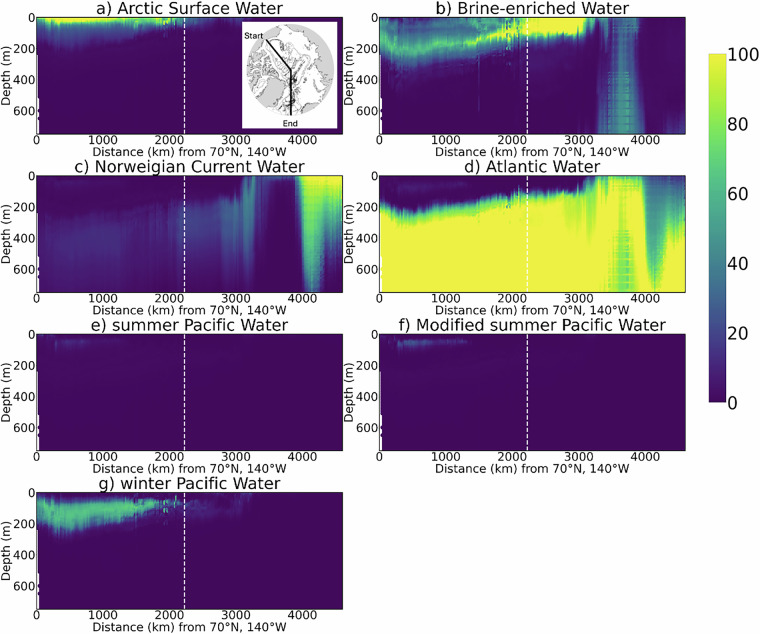
Latitudinal section of relative fractions of Arctic Ocean water masses (0-100%): (**a**) ASW, (**b**) BW, (**c**) NCW, (**d**) AW, (**e**) sPW, (**f**) MsPW, and (**g**) wPW. Section starts at the Canadian shelves at 70^∘^N, 140^∘^W (left) and ends at the Nordic Seas at 65^°^N, 0^°^E (right). Solid white lines in Fig. 5 in the main text mark the section location. Data are averaged into horizontal bins of 2.2 km and vertical bins of 10m for visualisation purposes.

**Fig. 5:**
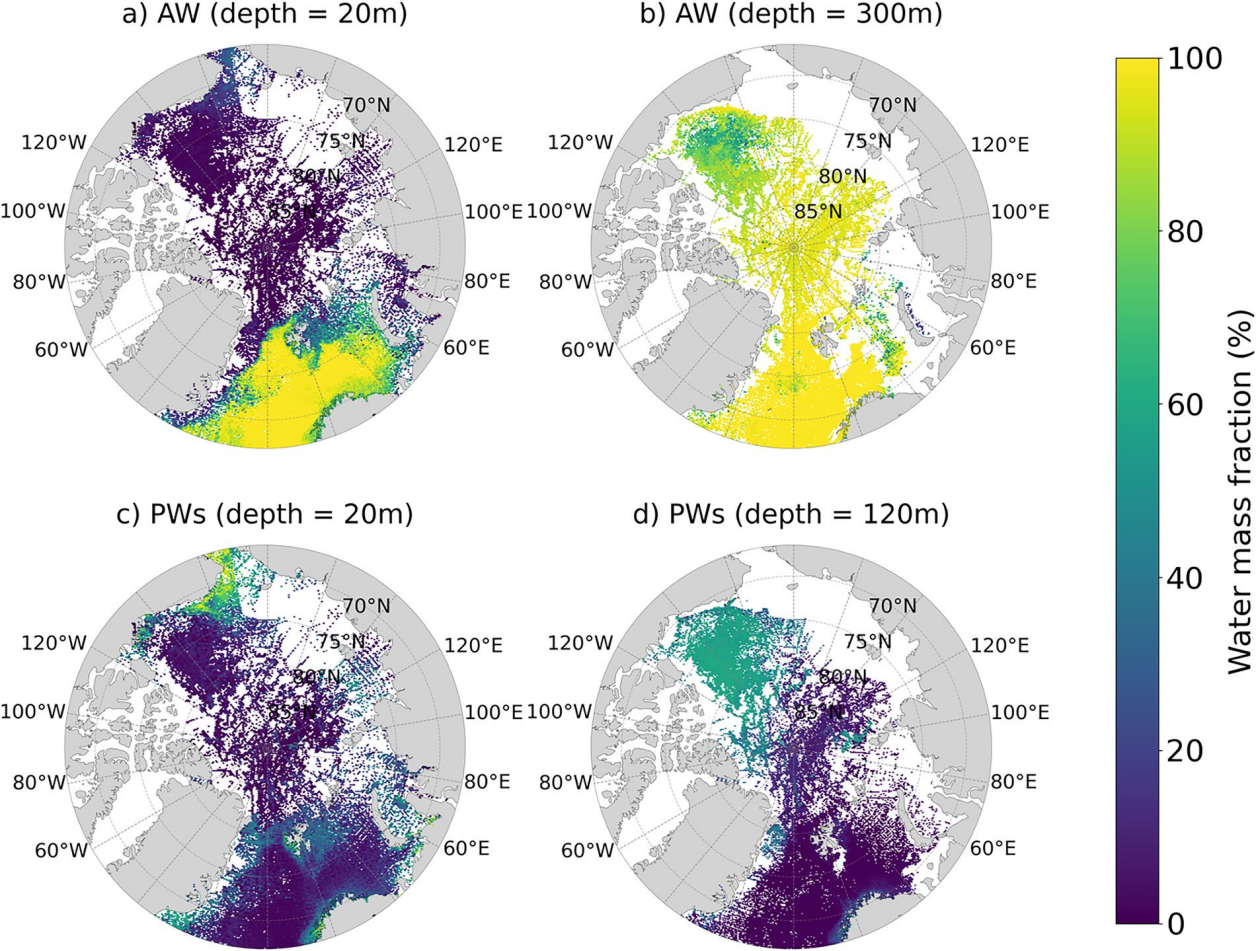
Maps of relative fractions of Arctic Ocean water masses (0-100%): (**a**) Atlantic Water (NCW + AW) at 20 m, (**b**) Atlantic Waters at 300m, (**c**) Pacific Waters (ACC + MsPW + wPW), at 20 m, and (**d**) Pacific Waters at 200 m. For clarity, data are averaged into horizontal bins of 2° longitude and 0.1° latitude and vertical bins of 10 m centered at the labeled depth.

We first outline the spatial distribution of AW (NCW +AW) and PW (sPW + wPW + MsPW), followed by a discussion of their respective flavours. The sites of AW and PW inflow into the Arctic Ocean^12,45,46^ are well reproduced by our dataset (Figs. 4 and 5). These inflow sites are the Fram Strait and Barents sea, where high AW fractions (>90%) are found, and the Bering Strait, where high PW fractions (>60%) are found. The key AW and PW pathways within the Arctic Ocean are also reproduced. Firstly, AW subduction below the lighter ASW and halocline Arctic waters (PW and BW)^12,45,46^, is represented by the deepening of the high AW layer (>90%) from the Fram Strait to the Canadian shelves (Fig. 4c) and moderate-high AW (>40%) at 300 m across the Arctic Ocean. Secondly, PW transport off the Chukchi Shelf into the western Arctic Ocean interior^12^ is represented by the high PW (>60%) in surface waters of the Chukchi Shelf and moderate PW (>40%) at 50-200 m in the Canada Basin. This PW distribution supports the notion of PW as the main source water of the Beaufort Gyre, which dominates the Canada Basin circulation at approximately 0-200 m^12^. Within the PW distribution, the wPW subtype is more prevalent than sPW and MsPW (Fig. 4). However, observational coverage is much sparser in regions of high sPW concentration near the Pacific gateway compared to the ITP sampling region where wPW dominates. Consequently, sPW is likely under-represented in our WMA dataset. For AW, NCW is dominant near the Atlantic gateway and is absent from the interior Arctic Ocean where modified Arctic AW dominates.

The formation and pathways of cold Arctic water masses^12,45^ are reproduced by this dataset (Fig. 5a,d). The formation of ASW (cold and fresh) and BW (cold and salty) via ice-ocean and air-ocean surface processes^45^ explains the high ASW and BW fractions (>60%) in the surface layer. Water density in the Arctic Ocean depends largely on salinity^12^, explaining the different pathways of ASW and BW: fresh ASW remains in the surface layer (0-50 m), while saltier BW sinks into the halocline (50-200 m)^47–49^ and, in some regions, to deeper waters (>500 m)^50^. Enrichment of BW in the eastern Arctic is consistent with established source regions of BW, particularly the Barents, Kara, Laptev and Siberian Shelf Seas, where enhanced sea ice production and coastal polynyas generate strong brine signals that ventilate the Arctic halocline^47–50^. Brine formed on the shelves can sink through convective and turbulent mixing and subsequently exported into the deep ocean basins^51–53^, potentially explaining the presence of BW (>30%) extending to 700 m in the Greenland Sea (3000-4000 km on Fig. 4b). This deep BW signature is consistent with the role of the Greenland Sea as a climate-critical region of deep convection and dense water production, facilitated by wintertime surface buoyancy loss, ice formation, and brine rejection^45,54–56^. The T-S characteristics of these BW signals, which align with previous literature, are provided in the Supplementary Information (Figure S7 and Text S4).

**Fig. 6:**
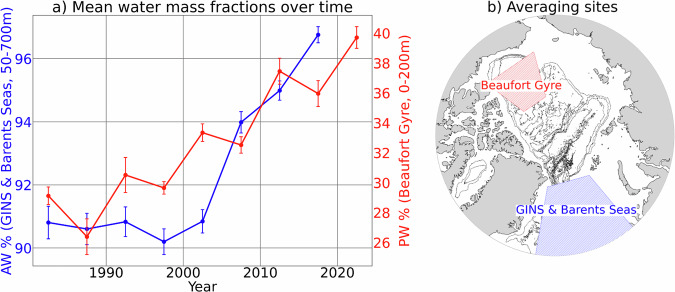
Scatterplot of 5-year mean percentages of Atlantic Water (NCW + AW, blue) and Pacific Water (sPW + MsPW + sPW, red) at (**b**) 0-200 m depth in two respective regions: the Beaufort Gyre and 50-700 m depth in the GINS and Barents Seas. These two regions were selected due to their notable data coverage since 1980 (Fig. 2 and Figures S1 and S2 Supplementary Information) and expectation that AW and PW are notable there. Error bars represent the standard deviation of the ensemble model’s predicted water mass fractions across five trained models, averaged over the same time and space.

Overall, our combined dataset of Arctic-wide water mass fractions agrees well with those previously reported in a number of different studies, capturing key characteristics of the known spatio-temporal distribution of Arctic water masses (Fig. 6).

Whilst our dataset captures the key features of Arctic oceanography, there are some regions where estimates deviate from past literature, likely due to methodological and data limitations. We find moderate-to-high PW (50–70%) in surface waters far from known PW inflow sites-including waters off east Greenland, Norway, and the Eurasian shelves, which are not generally reported as notable PW regions in the literature. We also find ASW confined to the top 25–50 m of the Canada Basin and BW present in surface waters of the Eurasian Basin, in contrast to earlier observations of ASW occupying the surface waters of both basins.

These differences are not unexpected given the methodological and data limitations, discussed in more detail in our sensitivity and uncertainty analyses (next sub-section). In particular, the assumption that water mass properties are conservative breaks down in the mixed layer where strong seasonal air-sea buoyancy fluxes modify CT and SA. When such modifications push properties outside the range of our defined SWTs, the classification becomes less effective. Our sensitivity tests suggest that these shallow water masses (ASW, PW, BW) are particularly affected by this, as their strong seasonal modification make them more difficult to distinguish than deeper waters. Further analysis at higher spatial and temporal scales is required to fully investigate these differences. Our current validation figures (Figs. 4 and 5), do not resolve seasonal variations in water mass distributions in our dataset. Seasonal processes, such as sea-ice melting and freezing and shear-driven mixing of surface waters^12^, can redistribute water masses seasonally, potentially contributing to the unexpected surface water features observed in our dataset. Additional factors contributing to elevated uncertainty in predictions include the close proximity of PW and BW in CT-SA space, as well as sampling biases-for example, a higher density of summer data when sea ice is reduced, which may also explain the thinner ASW layer than expected.

#### Temporal distribution

The Atlantification and Pacification of the Arctic Ocean, referring to the respective increase of AW and PW influence in the Arctic Ocean reported over the past few decades^12^ is broadly reproduced by this dataset. In the GINS and Barents Seas where AW enters the Arctic, the >5% increase in AW fraction Fig. 6 aligns with the well-reported, though still debated^57^, increase in AW volume and heat content entering the Arctic Ocean over the past few decades^19,20,58–60^. Reports of increasing AW influence in the Arctic focus on the Barents Sea and eastern Arctic, including a 8% y^−1^ increase in AW on the Barents Sea shelf from 1999 to 2020^60^. In the Beaufort Gyre (BG), the  ~10% increase in PW fraction Fig. 6 aligns with evidence of an increase in PW volume and heat content entering the Arctic Ocean over the past few decades^21,61^, including a near-doubling of the PW inflow through the Bering Strait from 2001-2014^62^ and a doubling of heat content of PW in the BG over the past 20 years^63–65^.

These literature-reported volume and temperature trends are useful to demonstrate consistency with fractional trends diagnosed in our dataset, but are not directly comparable. This is because a change in water mass fractions in a region is the result of (i) a genuine change in water mass volume, (ii) changes in source properties at formation, or (iii) a combination of both effects. The long-term increase in AW % occurs irrespective of whether the source water properties are defined using pre-2000 data or post-2010 (Figure S8 in Supplementary Information). Thus, we suggest that this AW fractional data captures the increasing volume of the Atlantic waters in the Arctic rather than purely changing properties of their unmixed, source properties. In contrast, the long-term increase in PW % diminishes when SWTs are defined using only pre-2000 data (Figure S8 in Supplementary Information). This suggests that the PW trend in the Beaufort Gyre is driven primarily by changing source properties rather than by an increase in the inflow volume of PW. These effects of long-term variations in SWT definitions, including the warming of AW and PW source waters, for our water mass estimates are discussed further in the following section. While fine-tuning the spatial boundaries of the GIN and Beaufort Gyre regions would be necessary for more robust temporal analyses, the goal here is not to explain these trends, but rather to demonstrate the utility of the dataset as a foundation for future research.

### Sensitivity of OMP output to SWT and weighting selection

**Fig. 7:**
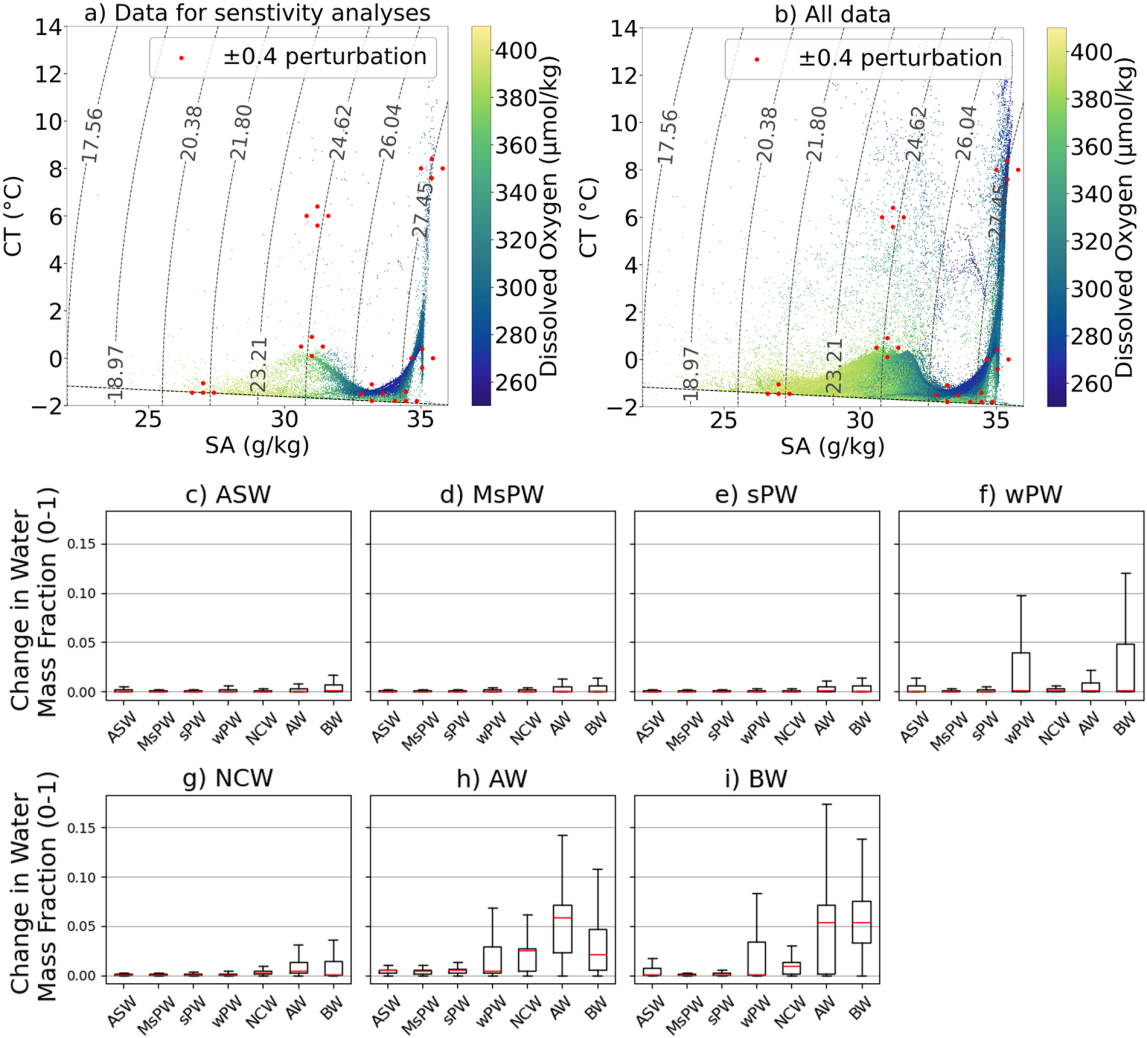
Sensitivity of OMP output to subjective SWT choice: (**a,b**) Red dots indicating perturbed properties of SWTs used in the 28 sensitivity tests overlay CT-SA diagrams of Arctic observational data compiled in this study, coloured by dissolved oxygen concentration (μmol/kg). Left plot shows the randomly sampled 10% subset of data used for the analyses, and representative of the full dataset (right plot). Measurements lacking oxygen data excluded. Dashed grey curved lines mark potential density referenced to 0 dbar (surface), and the black dashed horizontal line shows the freezing line. Boxplots (rows 2 and 3) show average resulting change in relative fractions of water masses (0-1) in response across the 4 perturbations of (**c**) ASW, (**d**) MsPW, (**e**) sPW, (**f**) wPW, (**g**) NCW, (**h**) AW, (**j**) BW. Red line indicates medium, and outliers are excluded.

First, we test the sensitivity of the OMP output to choice of SWT properties. CT and SA values of every SWTs are perturbed in 4 ways (±0.4CT and ±0.4 SA) and for every perturbation, the effect on the OMP output is quantified as the absolute change in relative water mass fractions (0-1). We report the results as box plots of the average absolute change across the 4 perturbations for each SWT (Fig. 7). A randomly sampled 10% subset of the data was used for the test, as demonstrated in Fig. 7. We chose ±0.4 because CT and SA extremes chosen following Steps 1-3 in Methods generally lie within these bounds. DO values of SWTs, which are weighted lower than CT and SA, were not altered. Any CT and SA that are perturbed below the freezing line are moved up to lie on the freezing line. The effect of these SWT perturbations is minimal for ASW, sPW, MsPW, and NCW (<0.05 change in fraction; Fig. 7), but larger for wPW, AW, and BW, for which the majority of values remain below 0.05, with upper tails reaching approximately 0.10 for wPW, 0.16 for AW, and 0.14 for BW (Fig. 7). The equivalent percentage changes are provided in Figure S9 in the Supplementary Information. This greater sensitivity is consistent with the close proximity of AW, wPW, and BW in CT-SA space, which makes them harder to distinguish. However, the overall influence of end member selection on OMP output – within a physically-reasonable parameter space – remains small.

**Fig. 8:**
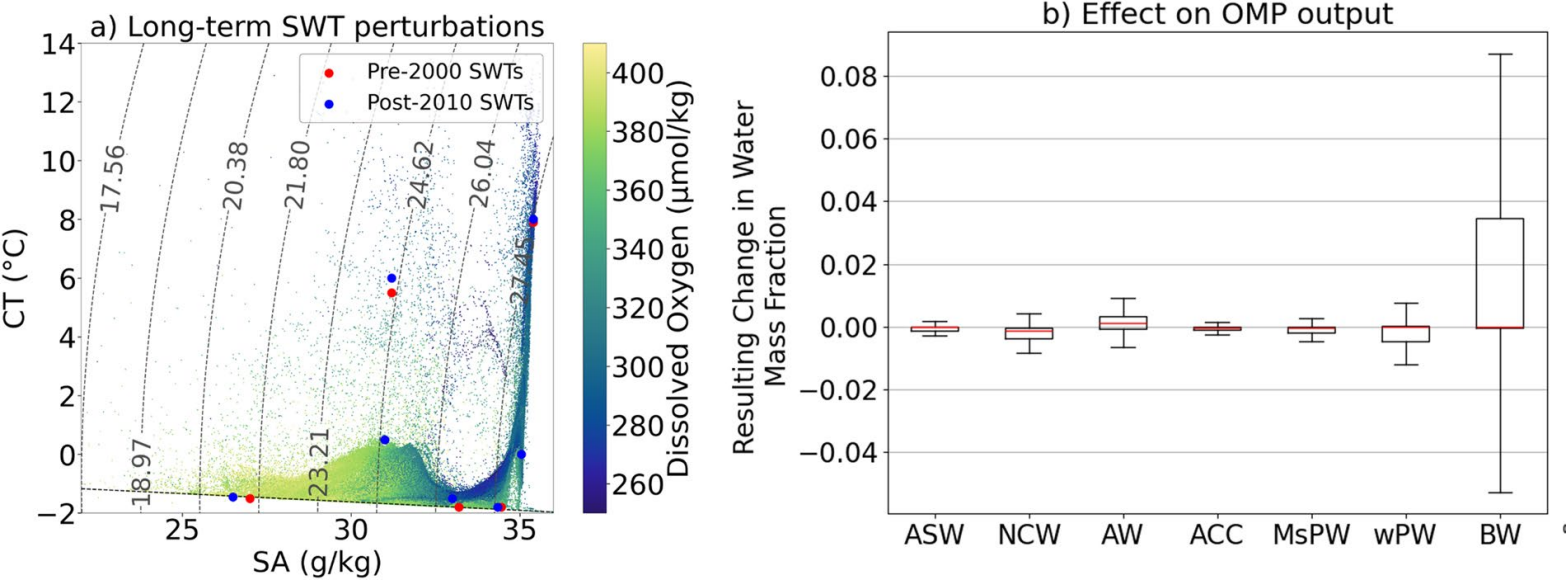
Sensitivity of OMP output to long-term changes in SWT properties: (**a**) CT-SA diagrams of Arctic observational data compiled in this study, coloured by dissolved oxygen concentration (*μ*mol/kg). Red and blue dots indicate SWT properties defined using Steps 1-3 in Methods for early (pre-2000) and late (post-2010) periods. (**b**) Resulting change in relative fractions of water masses (−1 to 1) in response to shifting SWTs from early to late period values. The test is applied to randomly sampled 10% subset of data. Not all SWT properties notably change between periods and oxygen properties of SWTs are not modified due to insufficient decadal coverage.

Second, we test the sensitivity of the OMP output to long-term modifications of SWT properties. SWTs are defined twice-first using post-2010 data and second using pre-2000 data following Steps 1-3 in Methods-and the effect on the OMP solution is quantified as the change in relative water mass fractions (ranging from −1 to 1). The test is applied to randomly sampled 10% subset of data. From pre-2000 to post-2010, we identify a warming of ASW, wPW, sPW, and NCW by 0.1, 0.4, 0.5, and 0.1 °C, respectively, and a freshening of ASW, wPW and BW by 0.1, 0.2 and 0.1 g/kg, respectively. Warming and freshening of Arctic water masses over recent decades is consistent with past literature^19–21,58,61^. Although other temporal changes in water mass properties have been documented (e.g., sPW deoxygenation^61^), they are not evident in our data compilation and/or cannot be identified here due to insufficient decadal coverage; for instance, oxygen changes in SWTs are not quantified in our test for this reason. Despite these property shifts, the effect on water mass fractions is negligible (<0.01; Fig. 8) for all water masses except BW, which shows a minor change (<0.08; Fig. 8). Given that BW is strongly modulated by seasonal sea-ice processes, this poorer constraint is not unexpected. We do not consider this long-term effect on our WMA dataset as fundamental given it’s basin-wide, long-term purpose.

**Fig. 9:**
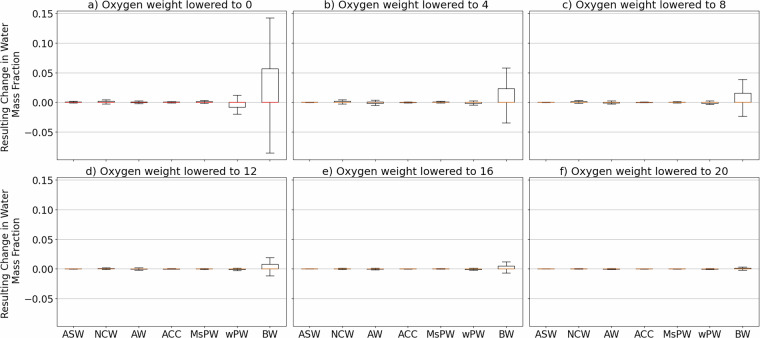
Resulting changes in relative water mass fractions (−1 to 1) are shown for a stepwise reduction of DO weight from 24 (equal to CT and SA) down to 0 in increments of 4. Each panel (**a–f**) shows the difference between the equal weight case (DO weight = 24) and one reduced-weight case (20, 16, 12, 8, 4, 0). The test is applied to a randomly sampled 10% subset of the data.

Finally, we test the sensitivity of OMP output to weighting choices. Following Lanham *et al*.^41^, DO is weighted 3 times smaller than CT and SA due to greater measurement uncertainties and non-conservative behaviour due to biological processes. We reduce the DO weight stepwise from 24 (equal to CT and SA) to 0, in increments of 4. Figure 9 Excluding BW and wPW, water masses remain insensitive to weighting changes. For wPW and BW, changes increase as DO weight is reduced, reaching -0.1 to 0.15 when DO is excluded entirely. Since DO helps separate these two water masses with similar CT-SA properties, this sensitivity is expected. Overall, all these water mass fractions changes are minimal. Thus, we do not consider choice of DO weighting to have a fundamental impact on our WMA dataset.

### Machine learning model metrics

**Fig. 10:**
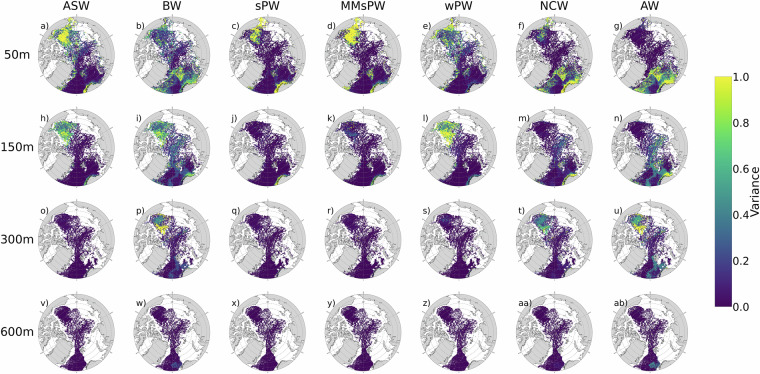
Normalised variance of the ensemble mean of relative fractions of Arctic Ocean water masses, indicating variability in predictions across 5 trained models. Water masses: Arctic Surface Water (ASW), Brine-enriched Waters (BW), summer Pacific Water (sPW), Modified summer Pacific Water (MsPW), winter Pacific Water (wPW), Norwegian Current Water (NCW), and Modified Atlantic Water (MAW). Variances are mapped for a range of depths: (**a–g**) 50 m, (**h–n**) 150 m, (**o–u**) 300 m, and (**v–ab**) 600 m. Values are averaged into horizontal bins of 2° longitude and 0.1° latitude and vertical bins of 10 m centered at the labeled depth.

Uncertainty of the ensemble mean of relative water mass fractions is assessed from the normalised variances across predictions from five trained models. Hotspots of high variance occur in near-surface waters across all water masses, especially ASW, sPW, and MsPW, and in halocline waters (150-300 m) for wPW, BW, and AW (Fig. 10). This variance increase may result from seawater properties lying outside the range of the training dataset-particularly in the mixed layer, where seasonal air-sea and ice-ocean fluxes modify CT and SA and make them non-conservative. In Arctic summer, these fluxes can warm and freshen ASW and sPW such that they fall outside the SWT definitions used in the OMP analysis to generate the training dataset. High variance may also result from high water mass mixing where seawater properties fall between multiple SWTs and classification is ambiguous. A notable example is the Beaufort Gyre halocline, where the properties of AW mix with the overlying BW and PWs. Limited data coverage, especially the scarcity of DO observations and spatio-temporal sampling biases also contribute to localized variance and reduced model accuracy.

The relative contribution of each input parameter to the model’s prediction of water mass fractions was quantified using the feature importance scores provided by the scikit-learn package^44^. The scores shows that SA dominates the prediction of relative Arctic water mass fractions, with CT contributing modestly. Other predictors, including depth, spatial coordinates, and seasonal terms, have minimal influence on the Arctic-wide model output (Figure S10 in Supporting Information) but may be important in specific regions and depths. This outcome is consistent with the physical understanding of Arctic Ocean stratification as being primarily controlled by salinity, with temperature serving as a secondary constraint^12^. Spatial and temporal predictors are likely to play a larger role in regions where CT and SA alone cannot distinguish between water masses, such as within the halocline where wPW and BW-which have similar CT and SA properties but differing DO-reside (Fig. 3).

**Fig. 11:**
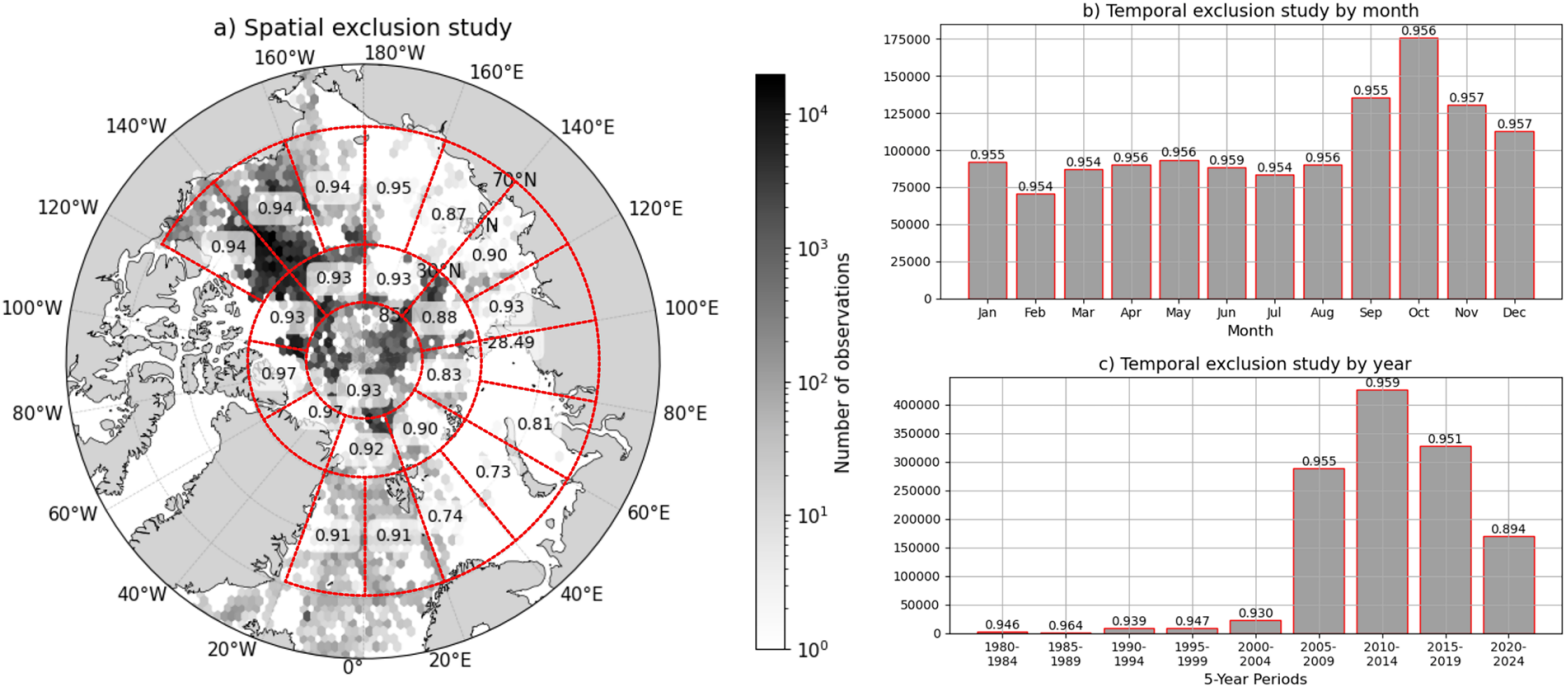
Spatial (**a**) and temporal (**b,c**) exclusion studies for the prediction of water masses in the Arctic region. Red lines mark subsets of data. The *R*^2^ value for each subset is calculated by excluding the subset during model training. The trained model is then used to predict water masses for the excluded region, and the *R*^2^ score is computed by comparing the predicted water masses with the true water masses. Temporal exclusion studies are performed by month (**b**) and year (**c**) separately.

To evaluate the robustness of the machine learning model, we conducted spatial and temporal exclusion studies. In these experiments, specific subsets of the data were withheld during training, and the trained model was then used to predict water mass fractions for the excluded subsets. Model performance was assessed using the *R*^2^ score, calculated by comparing predicted and observed water masses. Spatially (Fig. 11a), the *R*^2^ values remain consistently high (generally above 0.85) across different Arctic regions, indicating reliable predictive skill even when substantial spatial subsets are excluded from training. The lowest predictive skill, found in the Barents Sea and eastern Arctic (*R*^2^ values of 0.73–0.81), coincides with regions of low data availability. Temporally, the model also shows strong performance when evaluated by month (Fig. 11b), with *R*^2^ values ranging between 0.954 and 0.959, and by 5-year periods (Fig. 11c), with *R*^2^ values ranging from 0.894 to 0.964. Overall, these results confirm that the model maintains high predictive accuracy under both spatial and temporal exclusion, supporting its applicability for decadal- and basin-scale water mass classification.

### Validation of OMP analysis with objective classification

To objectively assess the water mass distributions generated by OMP analysis - which rely on external oceanographic knowledge and subjective criteria - we compare them to classes generated by a purely data-driven unsupervised ML approach. Specifically, we apply a Gaussian Mixture Model (GMM) to classify the CT, SA, and DO data without prior assumptions about water mass properties. Data points without DO measurements are excluded from the GMM classification. The GMM describes the multivariate structure of the dataset as a combination of overlapping Gaussian modes, whose means and internal spreads capture the characteristic properties and variability within the data. This technique has previously been shown to effectively identify water masses from in situ seawater observations^66–68^.

We determine 5 as the most appropriate *K* value using the Bayesian Information Criterion (BIC), Akaike Information Criterion (AIC), and the Silhouette (Si) score, following the methods of *K* selection by^66^. We use the scikit-learn ML library for Python^44^, and the code for the application of GMM in this paper is publicly available (see Code Availability section). Additional details on the GMM application including *K* selection and data bias mitigation are found in Text S2 in Supplementary Information. For completeness, results from a GMM classification with *K* value of 7-the number of water masses defined in our empirical study-are also included in Supplementary Information.

**Fig. 12:**
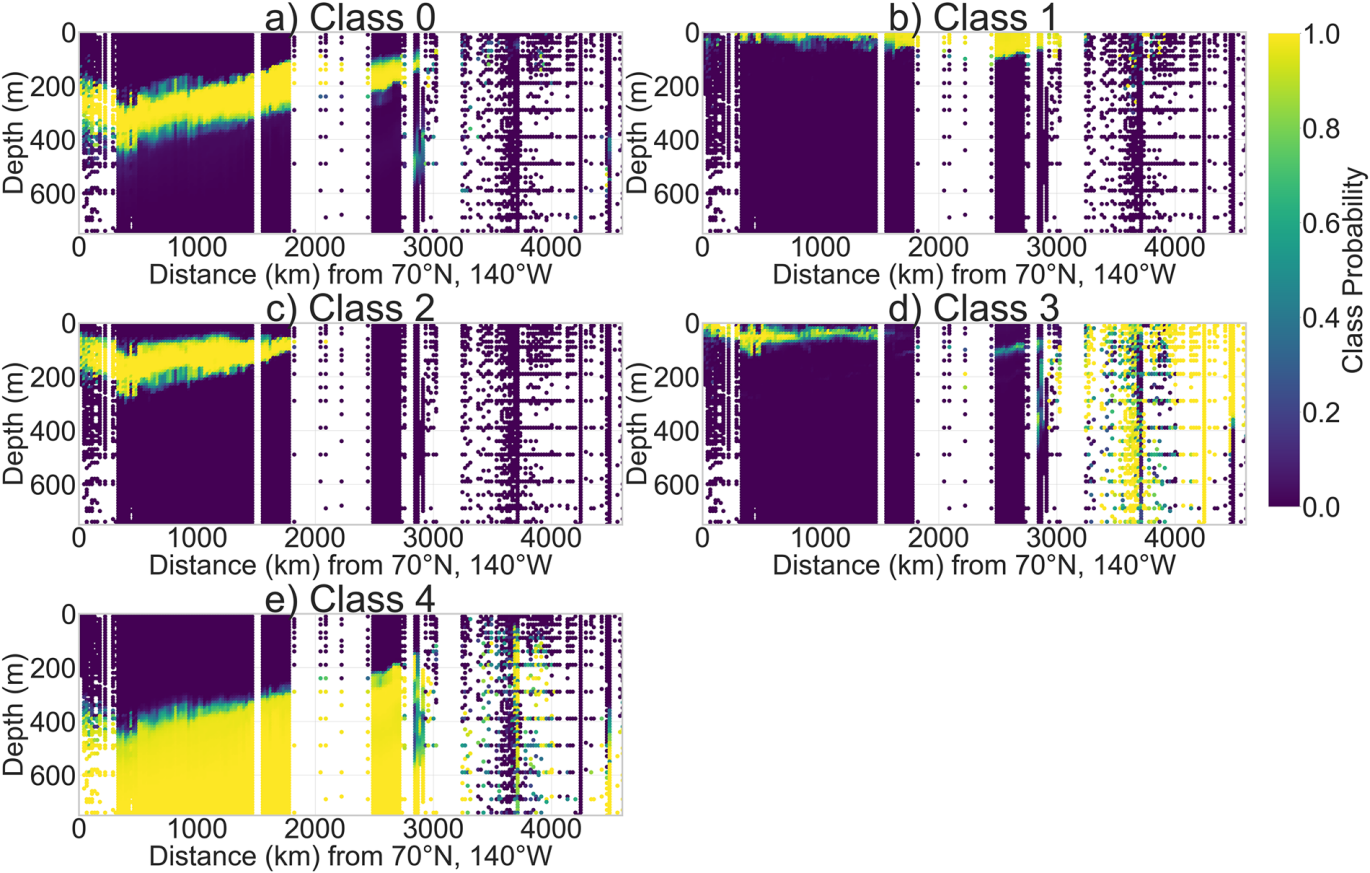
Latitudinal section of the probabilities generated by the GMM - representing the likelihood of each point to belong to each of the 5 classes: (**a–e**) Classes 0-4. Section starts at the Canadian shelves at 70°N, 140°W (left) and ends at the Nordic Seas at 65°N, 0°E (right). The black solid line on the bathymetric map in Fig. 4 marks the section location. Data are averaged into horizontal bins of 2.2km and vertical bins of 10m for visualisation purposes.

**Fig. 13:**
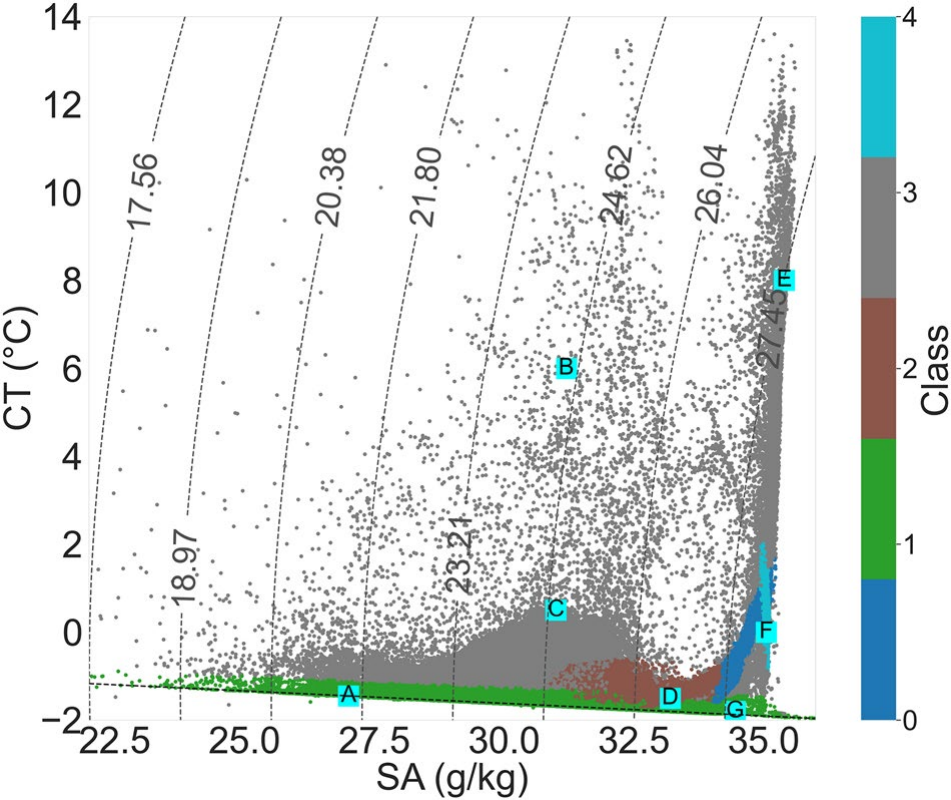
CT-SA diagram of all Arctic observational data compiled for this study, coloured by the dominant class (0-4) identified by the GMM. Observations without DO measurements are excluded. Cyan squares labels (**A–G**) denote the Source Water Types defined in our empirical study (see Table 2): A - ASW, B - sPW, C - MsPW, D - wPW, E - NCW), F - AW, and G - BW. Dashed curved contours mark potential density referenced to a pressure of 0 dbar (surface). The dashed horizontal line marks the freezing point of seawater.

The resulting spatial (Fig. 12) and CT-SA (Fig. 13) distributions of the GMM-derived classes shows broad similarities with the water masses identified by OMP analysis. We compare latitudinal sections of the GMM-derived class probabilities (Fig. 12) to that of our relative water mass fractions estimated via OMP analysis (Fig. 4 - contains OMP-derived water mass fractions as well as fractions predicted for data points without DO). Class 2 aligns closely in both spatial extent and CT-SA properties with wPW, residing at 50–200 m in the Canada Basin with unmixed properties of −1.50 °*C* and 33.20 g/kg. Class 1, characterised by low temperatures (<−1.0 °*C*) and variable salinity, overlaps with ASW and surface BW - the two cold surface water types identified in our OMP analysis. The remaining classes collectively capture the broad structure of Atlantic-origin waters. Class 0’s upper boundary tightly follows that of AW across the pan-Arctic section excluding in the GINS. Class 4 aligns well spatially and in CT-SA space with deeper, cooler AW layers. Class 3 coincides closely with NCW, the warmest AW flavour, in the eastern Arctic and GINS (>3000 km) and intersects with multiple near-surface (top 100m) water masses - including BW, ASW, sPW, and MsPW - in the western Arctic (<2000 km) with a broad spread of T and S properties.

Increasing the number of classes from 5-the statistically supported choice-to 7-the number of water masses defined in our empirical study-produces a broadly similar classification (Figure S13 and S14 in Supplementary Information). All classes remain, but Classes 3 and 4 are further sub-divided by temperature: Class 4 separates into younger, warmer AW in the eastern Arctic and older, cooler AW in the western Arctic, while Class 3 distinguishes NCW from a mixture of near-surface water masses in the western Arctic.

The agreement between the OMP and GMM classifications provides strong support for our empirical characterisation of Arctic water masses. Despite the subjective choices inherent in our classification -such as the selection of SWTs and their defining properties-the resulting water mass structure is not disimilar to that derived from an independent, largely objective GMM approach. That said, several limitations in the GMM application should be considered when interpreting its results. The algorithm places Gaussian components in regions of highest data density, which may reflect sampling biases rather than the true distribution of water mass properties. In particular, the observational data is strongly biased toward the summer months and ITP sampling regions, which reduces confidence in the robustness of the GMM results. These sampling biases remain even after basic subsampling (Text S2 in Supplementary Information). Additionally, some GMM classes, such as Class 1 and 3, have widely varying properties, suggesting insufficient sampling resolution to fully resolve the Arctic’s distinct water mass structure through purely data-driven approaches. These limitations highlight the continued importance of empirical classifications in data-sparse regions like the Arctic Ocean and the need to address key observational gaps. Future observing efforts, such as those planned for the International Polar Year 2032–2034^69^, could specifically target these under-sampled regions.

## Supplementary information

Supplementary Information

## Data Availability

The water mass dataset generated by this study, titled Water Masses of the Arctic (WMA), is publicly available through Figshare: 10.6084/m9.figshare.29646629^35^.

## Code availability

We provide the Python code as a collection of Jupyter notebooks to enable users to reproduce, extend, and apply the WMA dataset. The notebooks are available in a single GitHub repository (https://github.com/KateOglethorpe/WMA_classification) and archived on Zenodo^34^. The notebooks are succinctly described below:

• compiling_observations/level_1/: Process each data source, including loading and reformatting into standardized data frames containing relevant variables. Users are expected to use established, quality-controlled data products rather than raw sensor output.

• compiling_observations/level_2/: Compile all data sources, perform quality control, and remove Labrador Sea observations.

• wma_classification_tool/1_OMP_analysis/: Define SWTs, perform the OMP analysis, and evaluate the sensitivity of OMP solutions to SWT selection and weighting choices.

• wma_classification_tool/2_ML_extension/ML_extension.ipynb: Train a Random Forest 5 member model ensemble on the OMP-derived water mass fractions and use it to predict fractions at locations lacking DO data. The notebook also computes associated model uncertainties.

• wma_classification_tool/alternative_objective_clustering.ipynb: Apply a GMM to classify CT, SA, and DO data without prior assumptions about water mass properties.

• paper_figures/generate_paper_figures.ipynb: Reproduce the figures presented in this manuscript.

Together, this code allows users to fully reproduce the published WMA dataset and to generate updated or customised versions. For example, users can add new observations to the compilation dataset or redefine SWTs and re-run the classification procedure. The code also facilitates the generation of model-based WMA datasets. To illustrate this functionality, we provide a notebook that computes relative water mass fractions using CT and SA fields from the Arctic Subpolar Gyre sTate Estimate (ASTE)^70^, a dynamically consistent, observation-constrained model spanning 2002–2017. Comparing the observational WMA dataset with model-based WMA dataset enables evaluation of model skill in representing Arctic Ocean water masses and supports interpretation of future climate projections. For applications to model outputs that exhibit large known biases-for example, salinity biases common in ESMs^71^-users are encouraged to redefine SWTs and retrain the machine-learning model using the biased model’s CT-SA properties. This ensures that the classification remains physically consistent with the model’s water-mass structure.

## References

[1] Rantanen, M. *et al*. The arctic has warmed nearly four times faster than the globe since 1979. *Communications Earth & Environment* 3, August (2022).

[2] Meredith, M. *et al*. Polar regions. In Hans-Otto Pörtner, Debra C. Roberts, Valérie Masson-Delmotte, Panmao Zhai, Melinda Tignor, Elvira Poloczanska, Katja Mintenbeck, Aída Alegría, Marisol Nicolai, Andrew Okem, Jan Petzold, Belkacem Rama, and Nadine M. Weyer, editors, *IPCC Special Report on the Ocean and Cryosphere in a Changing Climate*, pages 203–320. Cambridge University Press, Cambridge, UK and New York, NY, USA (2019).

[3] Schweiger, A. *et al*. Uncertainty in modeled arctic sea ice volume. *Journal of Geophysical Research* **116**, C00D06 (2011).

[4] Meltofte, H. Biodiversity in the polar regions in a warming world. In *The Routledge Handbook of the Polar Regions*, pages 137–148. Routledge (2018).

[5] Siegert, M. *et al*. The arctic and the uk: Climate, research and engagement (2020).

[6] Rabe, B. *et al*. Arctic ocean basin liquid freshwater storage trend 1992–2012. *Geophys. Res. Lett.* **41**, 961–968 (2014).

[7] Haine, ThomasW. N. *et al*. Arctic freshwater export: Status, mechanisms, and prospects. *Global and Planetary Change* **125**, 13–35 (2015).

[8] Carmack, E. C. *et al*. Freshwater and its role in the arctic marine system: Sources, disposition, storage, export, and physical and biogeochemical consequences in the arctic and global oceans. *Journal of Geophysical Research: Biogeosciences* **121**, 675–717 (2016).

[9] Carmack, E. *et al*. Toward quantifying the increasing role of oceanic heat in sea ice loss in the new arctic. *Bull. Amer. Meteor. Soc.* **96**, 2079–2105 (2015).

[10] Good, P. *et al*. Recent progress in understanding climate thresholds: Ice sheets, the atlantic meridional overturning circulation, tropical forests and responses to ocean acidification. *Progress in Physical Geography: Earth and Environment* **42**, 24–60 (2018).

[11] Timmermans, Mary-Louise & Jayne, S. R. The Arctic Ocean Spices Up. *Journal of Physical Oceanography* **46**, 1277–1284 (2016).

[12] Timmermans, MaryLouise & Marshall, J. Understanding Arctic Ocean Circulation: A Review of Ocean Dynamics in a Changing Climate. *Journal of Geophysical Research: Oceans* **125**, e2018JC014378 (2020).

[13] Muilwijk, M., Smedsrud, L. H., Ilicak, M. & Drange, H. Atlantic water heat transport variability in the 20th century arctic ocean from a global ocean model and observations. *Journal of Geophysical Research: Oceans* **123**, 8159–8179 (2018).

[14] Timmermans, Mary-Louise The impact of stored solar heat on arctic sea ice growth. *Geophysical Research Letters* **42**, 6399–6406 (2015).

[15] Meyer, A., Fer, I., Sundfjord, A. & Peterson, A. K. Mixing rates and vertical heat fluxes north of Svalbard from Arctic winter to spring. *Journal of Geophysical Research: Oceans* **122**, 4569–4586 (2017).

[16] V. Polyakov, I. *et al*. Atlantification advances into the Amerasian Basin of the Arctic Ocean. *Science Advances* **11**, eadq7580 (2025). 39982990 10.1126/sciadv.adq7580 PMC11844727

[17] Muilwijk, M., Hattermann, T., Martin, T. & Granskog, M. A. Future sea ice weakening amplifies wind-driven trends in surface stress and arctic ocean spin-up. *Nature Communications* **15**, 6889 (2024). 39134517 10.1038/s41467-024-50874-0 PMC11319342

[18] Li, X. *et al*. Eddy activity in the arctic ocean projected to surge in a warming world. *Nature Climate Change* **14**, 156–162 (2024).

[19] Polyakov, I. V. *et al*. Greater role for Atlantic inflows on sea-ice loss in the Eurasian Basin of the Arctic Ocean. *Science* **356**, 6335 (2017). 10.1126/science.aai8204 28386025

[20] Richards, A. E., Johnson, H. L. & Lique, C. Spatial and Temporal Variability of Atlantic Water in the Arctic From 40 Years of Observations. *Journal of Geophysical Research: Oceans* **127**, e2021JC018358 (2022).

[21] A. Woodgate, R. & Peralta-Ferriz, C. Warming and Freshening of the Pacific Inflow to the Arctic From 1990-2019 Implying Dramatic Shoaling in Pacific Winter Water Ventilation of the Arctic Water Column. *Geophysical Research Letters* **48**, e2021GL092528 (2021).

[22] W. J. Emery. Water Types And Water Masses. In *E*ncyclopedia of Ocean Sciences, pages 3179–3187. Elsevier, (2001).

[23] Shrikumar, A., Lawrence, R. & Casciotti, K. L. PYOMPA version 0.3: Technical Note. *E*SS Open Archive, May Preprint, not peer-reviewed. https://essopenarchive.org/users/558299/articles/607576-pyompa-technical-note (2021).

[24] Oziel, L. *et al*. Climate change and terrigenous inputs decrease the efficiency of the future arctic ocean’s biological carbon pump. *Nature Climate Change* **15**, 171–179 (2025).

[25] Johnson, G. C. Quantifying Antarctic Bottom Water and North Atlantic Deep Water volumes. *Journal of Geophysical Research: Oceans* 113(C5), _eprint: 10.1029/2007JC004477 (2008).

[26] Liu, M. & Tanhua, T. Water masses in the Atlantic Ocean: Characteristics and distributions. *Ocean Science* **17**, 463–486 (2021).

[27] Jeansson, E., Olsen, A. & Jutterström, S. Arctic Intermediate Water in the Nordic Seas, 1991-2009. *Deep-Sea Research Part I: Oceanographic Research Papers* **128**, 82–97 (2017).

[28] Liguori, B. T. P., Ehlert, C. & Pahnke, K. The Influence of Water Mass Mixing and Particle Dissolution on the Silicon Cycle in the Central Arctic Ocean. *F*rontiers in Marine Science, 7, April Publisher: Frontiers Media S.A. (2020).

[29] Jeansson, E. *et al*. Sources to the east greenland current and its contribution to the denmark strait overflow. *Progress in Oceanography* **78**, 12–28 (2008).

[30] Lansard, B., Mucci, A., Miller, L. A., Macdonald, R. W. & Gratton, Y. Seasonal variability of water mass distribution in the southeastern beaufort sea determined by total alkalinity and *δ*18o. *Journal of Geophysical Research: Oceans* 117(C3), (2012).

[31] Lanham, J., Srinivasan, K., Cimoli, L. & Mashayek, A. Basin-wide Atlantic Ocean water mass classification and climatic variability from machine learning. *E*SSOAr preprint, 10.22541/essoar.176556325.59481750/v1 (2025).

[32] Lanham, J. *et al*. Observational evidence for a poleward migration of warm circumpolar deep water towards antarctica. *P*reprint. Submitted to Communications Earth & Environment, Available from: https://www.researchsquare.com/article/rs-7021596/v1 [Accessed 14 November 2025] (2025).

[33] Jackett, D. R. & McDougall, T. J. A neutral density variable for the world’s oceans - gibbs seawater (gsw) oceanographic toolbox. https://www.teos-10.org/software.htm (1997).

[34] Oglethorpe, K. *et al*. WMA_classification: Code for Water Masses of the Arctic dataset, v1.0.1. *Zenodo*10.5281/zenodo.17234455 (2025).

[35] Oglethorpe, K. *et al*. Water masses of the arctic from 40 years of hydrographic observations [dataset]. *figshare*10.6084/m9.figshare.29646629 (2025). 10.1038/s41597-026-06749-8 PMC13018476 41691005

[36] Lauvset, S. K. *et al*. Glodapv2.2022: the latest version of the global interior ocean biogeochemical data product [dataset]. *Earth System Science Data* **14**, 5543–5572 (2022).

[37] Behrendt, A., Sumata, H., Rabe, B. & Schauer, U. Udash - unified database for arctic and subarctic hydrography [dataset]. *Earth Syst. Sci. Data* **10**, 1119–1138 (2018).

[38] Rabe, B. *et al*. An assessment of Arctic Ocean freshwater content changes from the 1990s to the 2006–2008 period. *Deep-Sea Research Part I* **58**, 173–185 (2011).

[39] Gronell, A. & Wijffels, S. E. A semiautomated approach for quality controlling large historical ocean temperature archives. *Journal of Atmospheric and Oceanic Technology* **25**, 990–1003 (2008).

[40] Tomczak Jr, M. A multi-parameter extension of temperature/salinity diagram techniques for the analysis of non-isopycnal mixing. *Progress in Oceanography* **10**, 147–171 (1981).

[41] Lanham, J., Mazloff, M., Naveira Garabato, A. C., Siegert, M. & Mashayek, A. Seasonal regimes of warm circumpolar deep water intrusion toward antarctic ice shelves. *Communications Earth & Environment* **6**, 168 (2025). 40041764 10.1038/s43247-025-02091-w PMC11872733

[42] Falkner, KellyKenison *et al*. Dissolved oxygen extrema in the Arctic Ocean halocline from the North Pole to the Lincoln Sea. *Deep-Sea Research Part I: Oceanographic Research Papers* **52**, 1138–1154 (2005).

[43] Coachman, L. K., Aagaard, K. & Tripp, R. B. *Bering Strait: the regional physical oceanography*. University of Washington Press, (1975).

[44] Pedregosa, F. *et al*. Scikit-learn: Machine learning in python. *Journal of Machine Learning Research* **12**, 2825–2830 (2011).

[45] Talley, L. D., Pickard, G. L. & Emery, W. J. editors. *D*escriptive physical oceanography: an introduction. Academic Press, Amsterdam ; Boston, 6th ed edition, OCLC: ocn720651296. (2011).

[46] Rudels, B. Arctic Ocean circulation and variability - Advection and external forcing encounter constraints and local processes. *Ocean Science* **8**, 261–286 (2012).

[47] Cavalieri, D. J. & Martin, S. The contribution of alaskan, siberian, and canadian coastal polynyas to the cold halocline layer of the arctic ocean. *Journal of Geophysical Research: Oceans* **99**, 18343–18362 (1994).

[48] Bauch, D. *et al*. Impact of siberian coastal polynyas on shelf-derived arctic ocean halocline waters. *Journal of Geophysical Research: Oceans* 117(C9) (2012).

[49] Anderson, L. G. *et al*. Source and formation of the upper halocline of the arctic ocean. *Journal of Geophysical Research: Oceans* **118**, 410–421 (2013).

[50] Schauer, U., Muench, R. D., Rudels, B. & Timokhov, L. Impact of eastern arctic shelf waters on the nansen basin intermediate layers. *Journal of Geophysical Research: Oceans* **102**, 3371–3382 (1997).

[51] Schauer, U. The release of brine-enriched shelf water from storfjord into the norwegian sea. *Journal of Geophysical Research: Oceans* **100**, 16015–16028 (1995).

[52] Quadfasel, D., Rudels, B. & Selchow, S. The central bank vortex in the barents sea: watermass transformation and circulation. In *I*CES MSS Vol. 195: Hydrobiological variability in the ICES Area, 1980–1989, pages –. ICES, Copenhagen, Denmark, (1992).

[53] Pfirman, S. L., Bauch, D. & Gammelsrød, T. The northern barents sea: Water mass distribution and modification. In *P*roceedings of the AGU Geophysical Monograph Series, pages –. American Geophysical Union, Washington, D.C. (1994).

[54] Clarke, R. A., Swift, J. H., Reid, J. L. & Koltermann, K. P. The formation of greenland sea deep water: double diffusion or deep convection? *Deep Sea Research Part A. Oceanographic Research Papers* **37**, 1385–1424 (1990).

[55] Marshall, J. & Schott, F. Open-ocean convection: Observations, theory, and models. *Reviews of geophysics* **37**, 1–64 (1999).

[56] W. K. Moore, G., Våge, K., Pickart, R. S. & Renfrew, I. A. Decreasing intensity of open-ocean convection in the greenland and iceland seas. *Nat. Clim. Change* **5**, 877–882 (2015).

[57] Kong, B., Gao, L., Wang, S. & Guo, G. Cooling and sinking of the atlantic water in the eurasian basin since 1990s. *Geophysical Research Letters* **52**, e2025GL114720 (2025).

[58] Polyakov, I. V. *et al*. Weakening of Cold Halocline Layer Exposes Sea Ice to Oceanic Heat in the Eastern Arctic Ocean. *Journal of Climate* **33**, 8107 – 8123 (2020).

[59] Wang, Q. *et al*. Intensification of the atlantic water supply to the arctic ocean through fram strait induced by arctic sea ice decline. *Geophysical Research Letters* **47**, e2019GL086682 (2020).

[60] Strzelewicz, A., Przyborska, A. & Walczowski, W. Increased presence of atlantic water on the shelf south-west of spitsbergen with implications for the arctic fjord hornsund. *Progress in Oceanography* **200**, 102714 (2022).

[61] Arroyo, A., Timmermans, Mary-Louise, Le Bras, I., Williams, W. & Zimmermann, S. Declining o2 in the canada basin halocline consistent with physical and biogeochemical effects of pacific summer water warming. *Journal of Geophysical Research: Oceans* **128**, e2022JC019418 (2023).

[62] Woodgate, R. A. Increases in the pacific inflow to the arctic from 1990 to 2015, and insights into seasonal trends and driving mechanisms from year-round bering strait mooring data. *Progress in Oceanography* **160**, 124–154 (2018).

[63] Timmermans, Mary-Louise, Toole, J. & Krishfield, R. Warming of the interior arctic ocean linked to sea ice losses at the basin margins. *Science advances* **4**, eaat6773 (2018). 30167462 10.1126/sciadv.aat6773 PMC6114986

[64] Timmermans, Mary-Louise & Toole, J. M. The arctic ocean’s beaufort gyre. *Annual Review of Marine Science* **15**, 223–248 (2023). 35973719 10.1146/annurev-marine-032122-012034

[65] Timmermans, M.-L. *et al*. Introduction to the special collection on the arctic ocean’s changing beaufort gyre, (2025).

[66] Zheng, P. Clustering under the ice. Msc dissertation, University of Cambridge, Supervised by Emma Boland (2023).

[67] Jones, D. C., Holt, H. J., Meijers, AndrewJ. S. & Shuckburgh, E. Unsupervised Clustering of Southern Ocean Argo Float Temperature Profiles. *Journal of Geophysical Research: Oceans* **124**, 390–402 (2019).

[68] Maze, G. *et al*. Coherent heat patterns revealed by unsupervised classification of argo temperature profiles in the north atlantic ocean. *Progress in Oceanography* **151**, 275–292 (2017).

[69] International Polar Year 5. Ipy-5: The fifth international polar year (2032-2033), Accessed: 2025-07-15 (2025).

[70] Nguyen, A. T. *et al*. The arctic subpolar gyre state estimate: Description and assessment of a data-constrained, dynamically consistent ocean-sea ice estimate for 2002-2017. *Journal of Advances in Modeling Earth Systems* **13**, e2020MS002398 (2021).

[71] Khosravi, N. *et al*. The arctic ocean in cmip6 models: Biases and projected changes in temperature and salinity. *Earth’s Future* **10**, e2021EF002282 (2022).

[72] Toole, J. M., Krishfield, R. A., Timmermans, MaryLouise & Proshutinsky, A. The Ice-Tethered profiler: ArgoEuroArgoDataSelection of the Arctic. *Oceanography* **24**, 126–135 (2011).

[73] Euro-Argo. Euro-argo data selection tool [dataset], Accessed: 2025-02-21 (2025).

[74] Tippenhauer, S. *et al*. Physical oceanography based on ocean city ctd during polarstern cruise ps122 [dataset] (2023).

[75] Rudels, B. *et al*. The interaction between waters from the arctic ocean and the nordic seas north of fram strait and along the east greenland current: results from the arctic ocean-02 oden expedition. *J. Mar. Syst.* **55**, 1–30 (2005).

[76] Aksenov, Y., Bacon, S., Coward, A. C. & Holliday, N. P. Polar outflow from the Arctic Ocean: A high resolution model study. *Journal of Marine Systems* **83**, 14–37 (2010).

[77] Timmermans, Mary-Louise, Krishfield, R., Laney, S. & Toole, J. Ice-tethered profiler measurements of dissolved oxygen under permanent ice cover in the arctic ocean. *J. Atmos. Oceanic Technol.* **27**, 1936–1949 (2010).

[78] Steele, M. *et al*. Circulation of summer Pacific halocline water in the Arctic Ocean. *Journal of Geophysical Research: Oceans* 109(2), February Publisher: Blackwell Publishing Ltd. (2004).

[79] Timmermans, Mary-Louise *et al*. Mechanisms of pacific summer water variability in the arctic’s central canada basin. *J. Geophys. Res. Oceans* **119**, 7523–7548 (2014).

[80] Nikolopoulos, A. *et al*. The western arctic boundary current at 152 w: Structure, variability, and transport. *Deep Sea Research Part II: Topical Studies in Oceanography* **56**, 1164–1181 (2009).

[81] Planat, Noémie, Tremblay, L. B., Dufour, C. O. & Straub, D. Seasonal and decadal geostrophic pathways of pacific and atlantic waters in the arctic amerasian basin from observations. *Journal of Geophysical Research: Oceans* **130**, e2024JC021560 (2025).

[82] MacKinnon, J. A. *et al*. A warm jet in a cold ocean. *Nature communications* **12**, 2418 (2021). 33893280 10.1038/s41467-021-22505-5 PMC8065036

[83] Pemberton, P., Nilsson, J., Hieronymus, M. & Markus Meier, H. E. Arctic ocean water mass transformation in S-T coordinates. *Journal of Physical Oceanography* **45**, 1025–1050 (2015).

[84] Tsubouchi, T. *et al*. The Arctic Ocean in summer: A quasi-synoptic inverse estimate of boundary fluxes and water mass transformation. *Journal of Geophysical Research: Oceans* 117(1) (2012).

[85] Skogseth, R., Haugan, P. M. & Jakobsson, M. Watermass transformations in storfjorden. *Continental Shelf Research* **25**, 667–695 (2005).

[86] Pawlowicz, R., J. McDougall, T., Feistel, R. & Tailleux, R. émi An historical perspective on the development of the thermodynamic equation of seawater-2010. *Ocean Science* **8**, 161–174 (2012).

