# Supplementary material for: Water Masses of the Arctic from 40 Years of Hydrographic Observations
Source: Sci Data. 2026 Feb 14;13:456. doi: 10.1038/s41597-026-06749-8 (PMC13018476; doi:10.1038/s41597-026-06749-8)
Supplement: Supplementary file 1 — Supplementary Information [file 41597_2026_6749_MOESM1_ESM.pdf]

Supplementary Information

Water Masses of the Arctic from 40 Years of Hydrographic Observations

Contents

1. Text S1 to S3 1

2. Figures S1 to S14 2

1 Supplementary Text

Text S1: Residuals of the OMP solution

Figure S5 shows residuals in the OMP solution for a range of depths. Most data points in the solution show residuals close to zero, indicating that the observed conservative temperature (CT), absolute salinity (SA), and dissolved oxygen (DO) are well explained as linear mixtures of the selected Source Water Types (SWTs). This confirms that the chosen SWTs capture the dominant water mass structure of the top 1km of the Arctic Ocean. The largest residuals are found for CT and SA at near-surface waters in the central Arctic Ocean and throughout the water column in the Greenland-Iceland-Norwegian Seas (GINS), south of the Fram Strait. We interpret these areas as regions in which CT and SA are out of range of the selected Source Water Types (SWTs), suggesting that there are un-modeled water masses here. For the near-surface waters and open-ocean convection regions in the GINS, this is expected given there are large air-sea and ice-sea buoyancy fluxes and thus CT and SA properties are not conserved. Un-modelled water mass in the GINS may also arise because our SWTs were selected to constrain the properties of central Arctic water masses, leading to a less accurate representation of water mass varieties in the GINS.

Text S2: Objective validation of water mass classification

We apply a Gaussian Mixture Model (GMM) to objectively classify the CT, SA, and dissolved oxygen (DO) data. GMM training, including selection of the number of classes, K, was performed on a subset of the data in which spatial and temporal biases had been partially mitigated. The trained GMM was then used to predict the likelihood of belong to each class for the full dataset.

Because the GMM relies on fitting probability density functions, it is more sensitive to uneven spatial and temporal sampling than OMP, where each sample is independently fit to the SWTs. To reduce this bias, we subsampled only Ice-Tethered Profiler (ITP) data, which disproportionately contributed to both the spatial bias toward the Canada Basin and the interannual bias toward the last decade. Other data sources were too sparse to justify subsampling. To limit summer bias, we randomly sampled 1,500 ITP profiles from each month. To reduce spatial bias, we grouped ITP profiles into 10° longitude $\times$ 2° latitude bins, with a maximum sampling density of 0.008 profiles per km^2 per bin. This procedure substantially reduced the spatial and temporal biases of the combined dataset (including all data sources) yet notable biases persisted in the training subset (Figure S12).

The number of classes, K, is the only free parameter of the GMM. Following [1], we determined K using the Bayesian Information Criterion (BIC), Akaike Information Criterion (AIC), and Silhouette score (Si). BIC and AIC quantify the trade-off between model fit and complexity, while Si assesses cluster separation and cohesion. Figure S11 shows BIC, AIC, and Si values across different K. We selected $K =$ 5 as the most appropriate value, as it marks the statistical "elbow" in the AIC and BIC curves, capturing key data structure without the oversimplification implied by higher Si scores at lower K.

Text S3: Properties of brine-enriched waters

Given the spatial complexity of the distribution of brine-enriched waters (BW), we offer further details on its pathways and T-S characteristics. As described in the main text, high concentrations of BW in the eastern Arctic are consistent with established source regions of BW on the Arctic shelves where enhanced sea ice production and coastal polynyas generate strong brine signals that ventilate the Arctic halocline [2, 3, 4, 5]. Moderate BW signals in intermediate waters of the Greenland Sea are also consistent with deep wintertime convection in the region [6, 7, 8, 9], and BW drainage off continental slopes into deep basins [10, 11, 12]. T-S characteristics of BW further support these pathways. The saltiest BW (>34.9; red box in Figure S7) are found at sites of intense sea ice formation and brine release (inset map in Figure S7), for example Storfjorden—a large, recurrent latent heat polynya south-east of the Svalbard Archipelago [13, 10]. During winter in Storfjorden, northeasterly winds open the polynya, driving extensive ice production and injecting brine into the underlying water column. The salinity of BW formed in Storfjorden has previously been reported to range from 34.8 to 35.1 [10, 14], consistent with the salinity characteristics of BW identified in our analysis.

2 Supplementary Figures

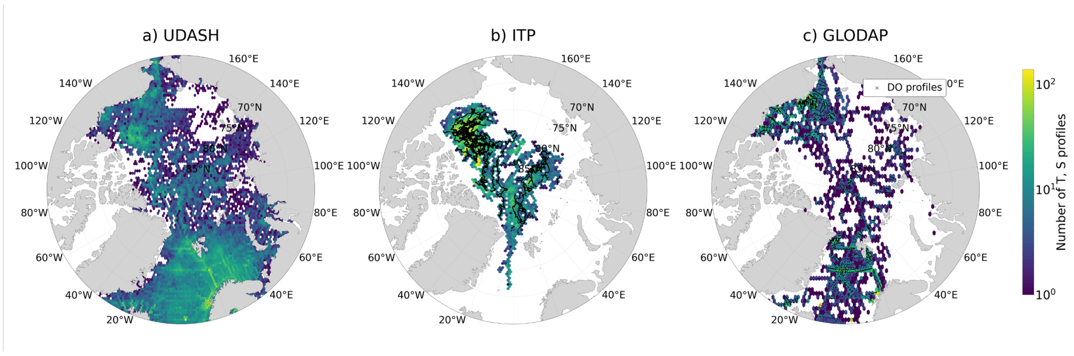

Figure 1: Spatial distributions of Arctic Ocean hydrographic observations from the three primary data sources used in this study: a) Unified Database for Arctic and Subarctic Hydrography (UDASH) [15], b) Ice-Tethered Profilers (ITP) [16], and c) Global Ocean Data Analysis Project (GLODAP) [17]. Hexagons are coloured by number of T, S profiles per bin where each bin covers approximately 3.6° in longitude and 0.25° in latitude. Black crosses mark the sites where DO is measured.

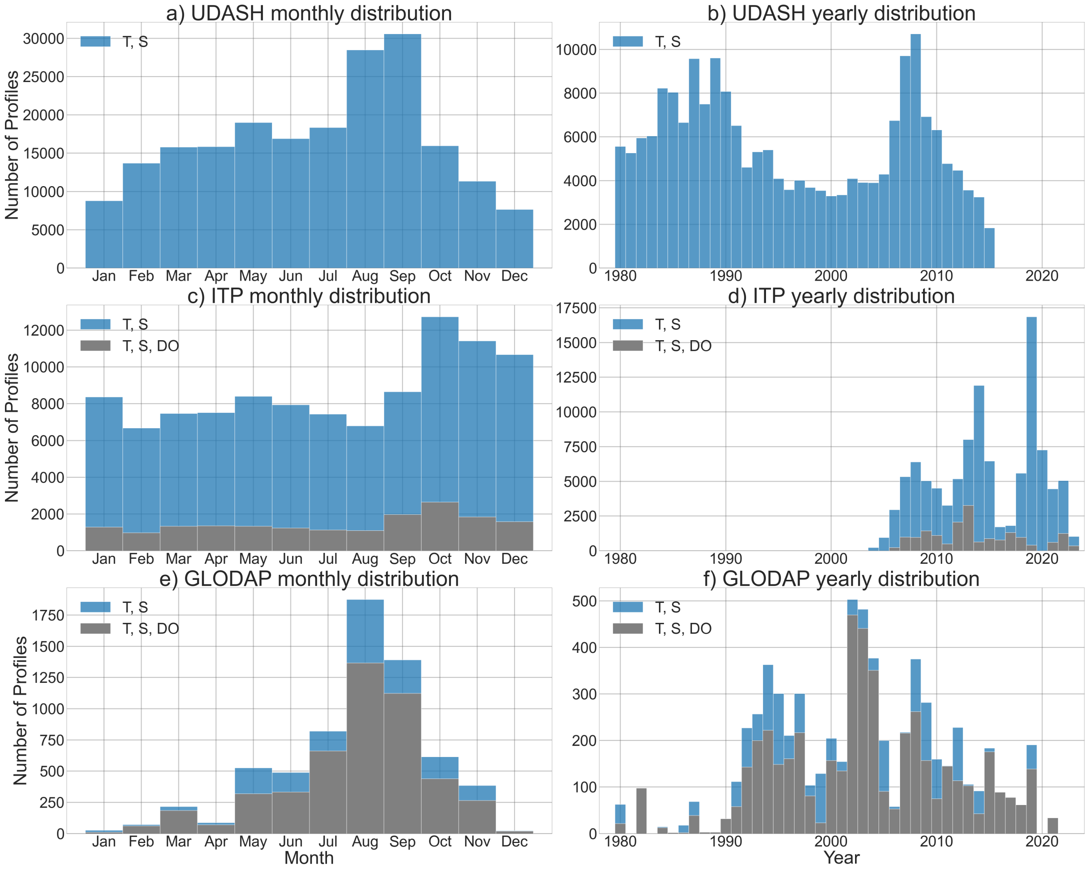

Figure 2: Monthly (left column) and yearly (right column) distributions Arctic Ocean hydrographic observations from the three primary data sources used in this study: a-b) Unified Database for Arctic and Subarctic Hydrography (UDASH) [15], c-d) Ice-Tethered Profilers (ITP) [16], and e-f) Global Ocean Data Analysis Project (GLODAP) [17]. Grey bars mark profiles when DO is measured.

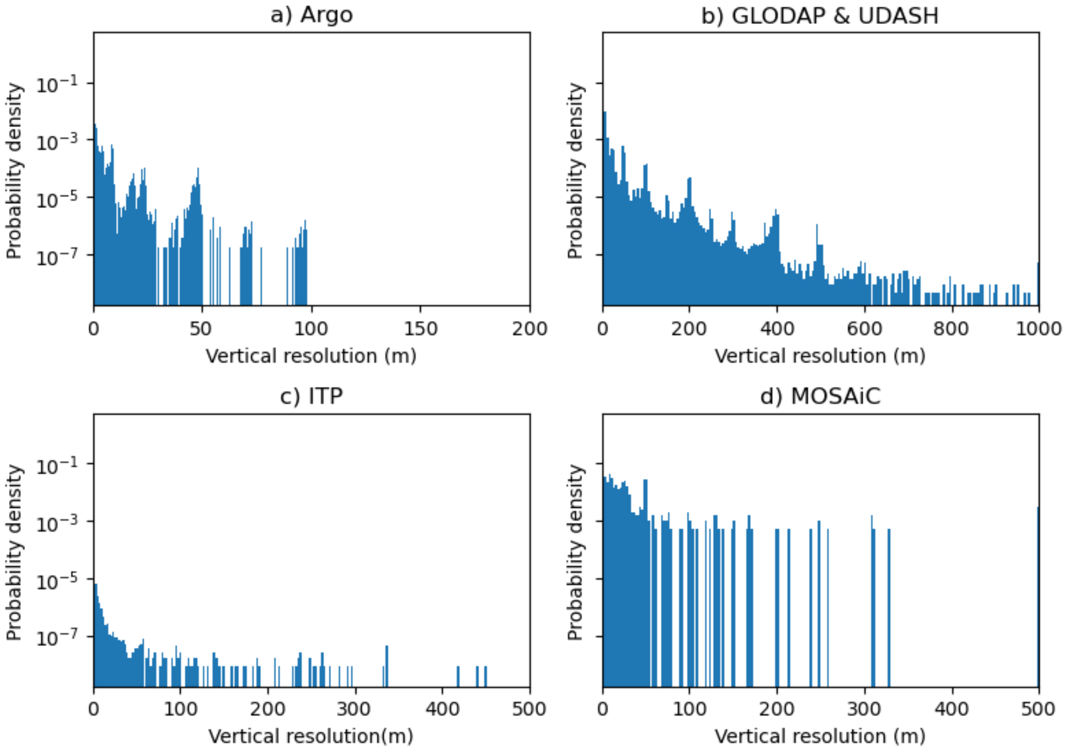

Figure 3: Probability density distributions of vertical resolution of data sources prior to averaging vertically into 10m bins: a) Argo, b) Unified Database for Arctic and Subarctic Hydrography (UDASH) and Global Ocean Data Analysis Project (GLODAP), c) Ice-Tethered Profilers (ITP), and d) MOSAiC. Probability densities are shown on a logarithmic scale. Horizontal axes differ for each data source.

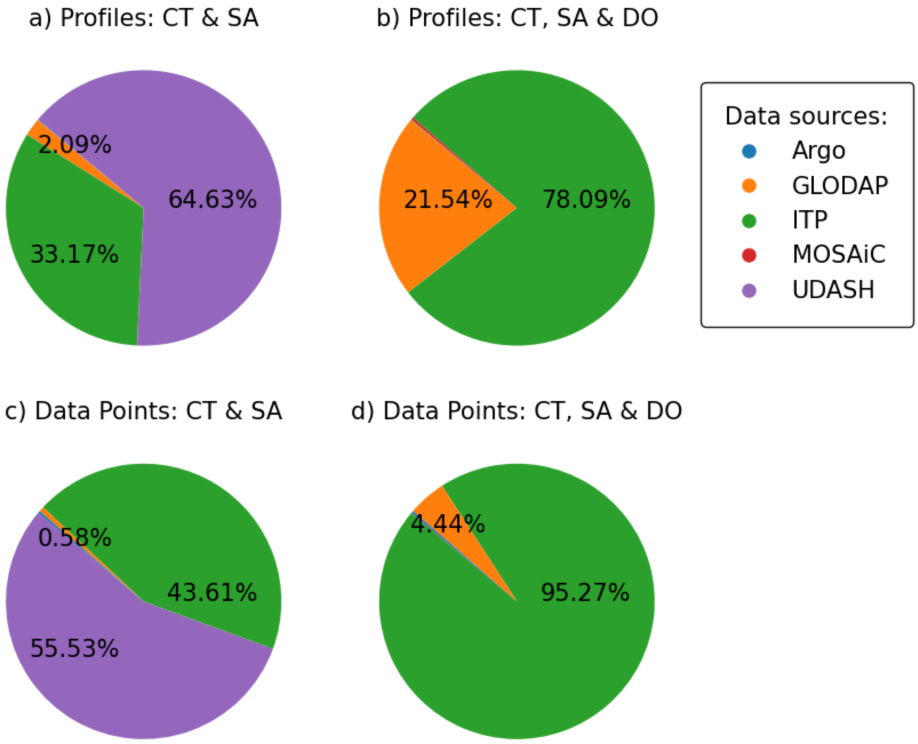

Figure 4: Relative contributions of each data source to the compiled Arctic observational dataset, expressed as percentages (0–100%), calculated in four ways: a) number of CT and SA profiles; b) number of CT, SA, and DO profiles (excluding profiles without DO); c) number of CT and SA data points; and d) number of CT, SA, and DO data points (excluding data points without DO). See Table 2 for data sources information. Text labels indicate percentage contributions from the dominant sources—ITP, UDASH, and GLODAP. Note that UDASH does not include DO measurements.

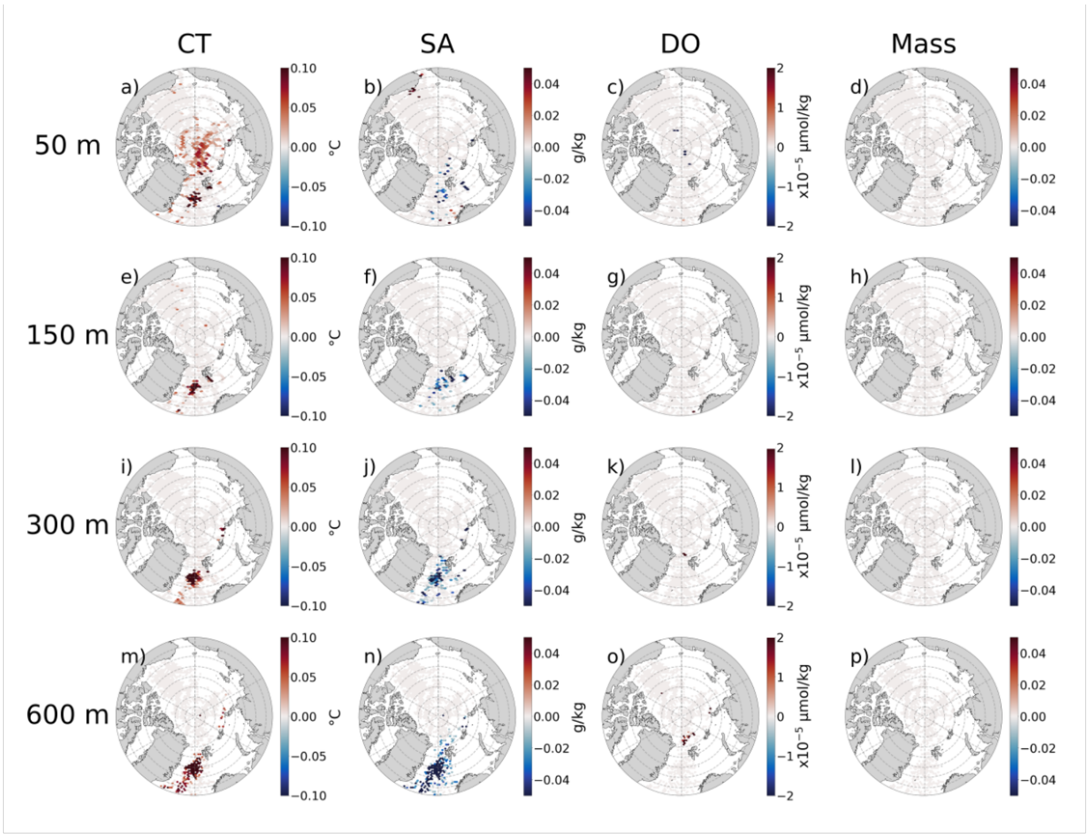

Figure 5: Residuals for all four variables in the water mass classification solution: conservative temperature (CT), absolute salinity (SA), dissolved oxygen (DO), and mass. Residuals are mapped for a range of depths: a-d) 20m, e-h) 120m, i-l) 300m, and m-p) 600m. Values are averaged into horizontal bins of 2° longitude and 0.1° latitude and vertical bins of 10m centered at the labeled depth.

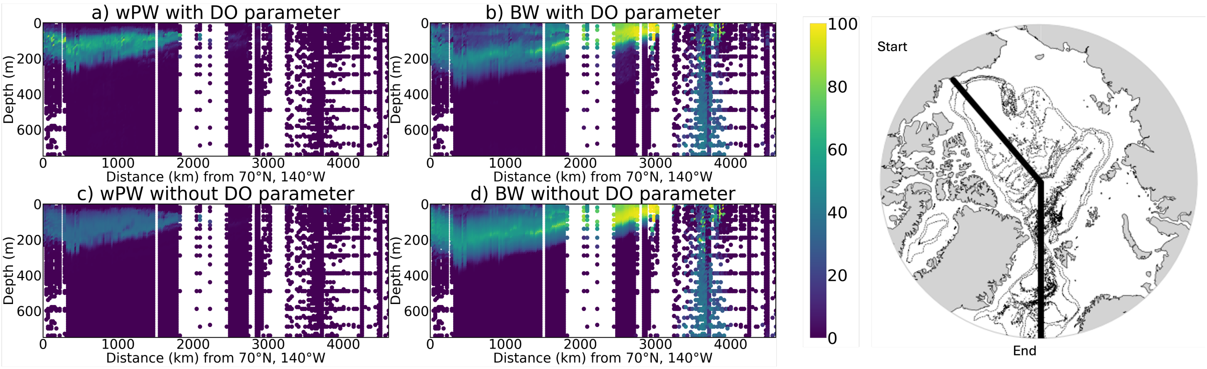

Figure 6: Sections of relative water mass fractions of winter Pacific Water (wPW) and Brine-enriched Water (BW) estimated via OMP analysis where DO concentration is included (top row) and excluded (bottom row). Excluding DO from the OMP analysis results in up to a 50% reduction in the estimated wPW relative fractions in the upper 250m of the Canada Basin.

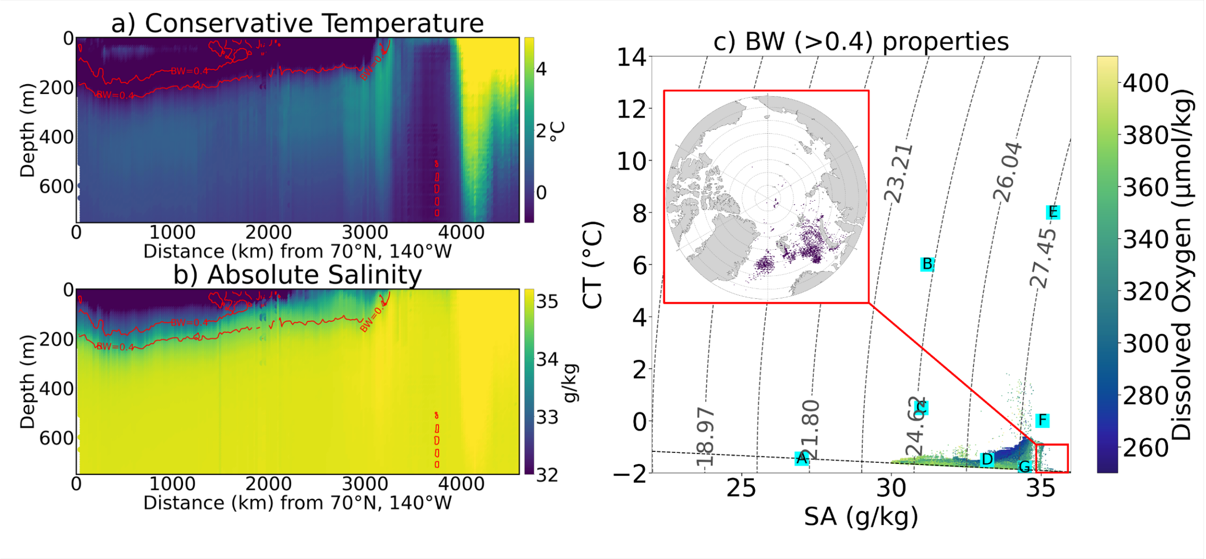

Figure 7: Latitudinal section of a) Conservative Temperature and b) Absolute Salinity, with the 40% Brine-enriched Water (BW) fraction contour overlain in red. Section starts at the Canadian shelves at 70°N, 140°W (left) and ends in the Nordic Seas at 65°N, 0°E (right). Data are averaged into horizontal bins of 2.2 km and vertical bins of 10 m for visualization. Panel c) shows the T–S distribution of BW (>40% fraction), coloured by dissolved oxygen, with σ_0 density contours overlain. Cyan squares mark the selected source water types (SWTs) A–G used in the analysis. The inset map indicates the geographic locations of the saltiest BW (>35.9 g/kg) marked by a red box.

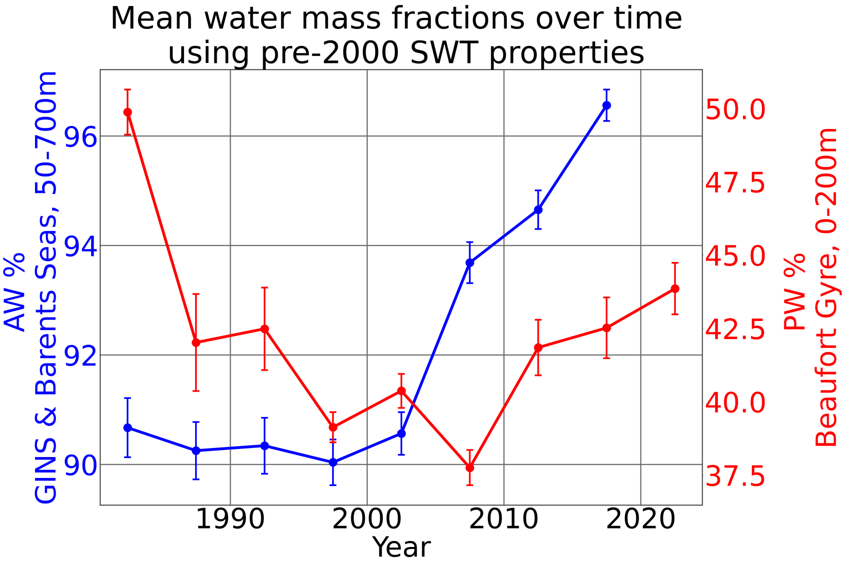

Figure 8: Scatterplot of 5-year mean percentages of Atlantic Water (AW, blue) and Pacific Water (PW, red) at b) 0–200 m depth in two respective regions: the Beaufort Gyre and 50–700 m depth in the GINS and Barents Seas. The properties of unmixed water masses used to estimate AW and PW percentages in this plot are defined using pre-2000 data, which differs to those used to estimate AW and PW percentages in Figure 6 (main text) where data spanning 1980 to 2024 were used.

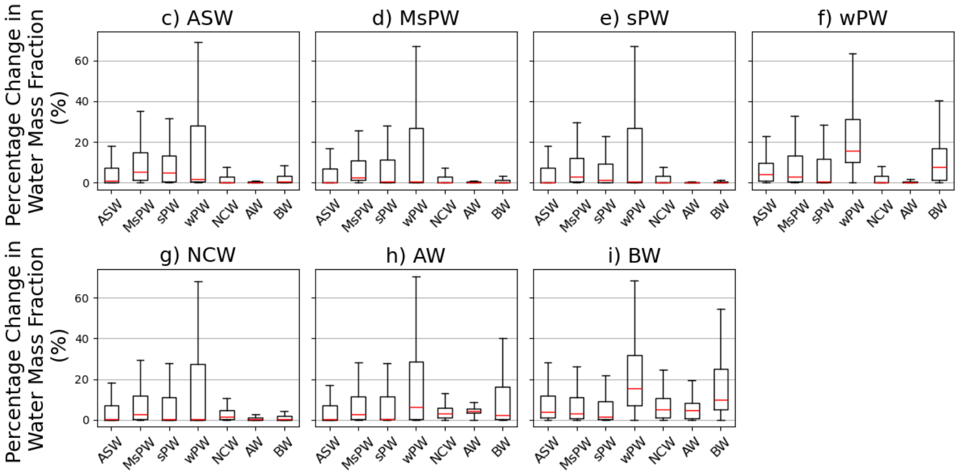

Figure 9: Sensitivity of OMP output to subjective SWT choices. Boxplots (rows 2 and 3) show average resulting percentage in relative fractions of water masses (0-100%) in response across the 4 perturbations of c) ASW, d) MsPW, e) sPW, f) wPW, g) NCW, h) AW, j) BW. Red line indicates medium, and outliers are excluded. Small relative fractions of <0.05 were excluded.

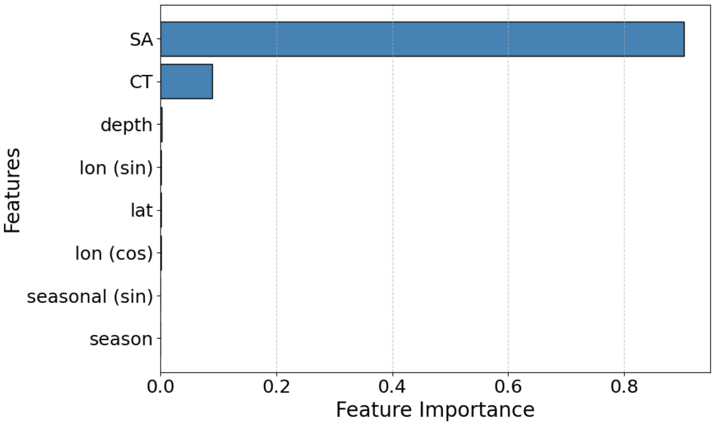

Figure 10: Feature importance of predictors used in the random forest ensemble used in this study for predicting Arctic water mass fractions from CT and SA observations where DO measurements are unavailable. Additional contextual features—including longitude (sin), longitude (cos), latitude, depth, season (sin) and month—are included.

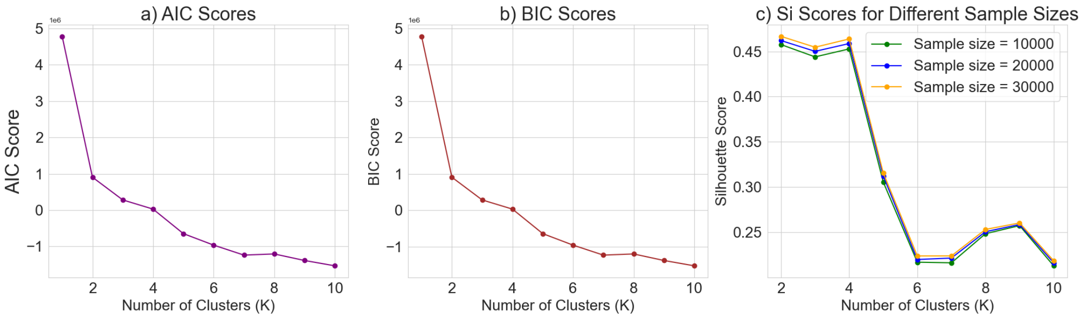

Figure 11: Metrics used to determine the number of classes, K, which determines the number of mixture components in the Gaussian Mixture Model: a) Akaike Information Criterion (AIC), Bayesian Information Criterion (BIC), and c) the Silhouette score (Si). Metrics are plotted for K values 2-10.

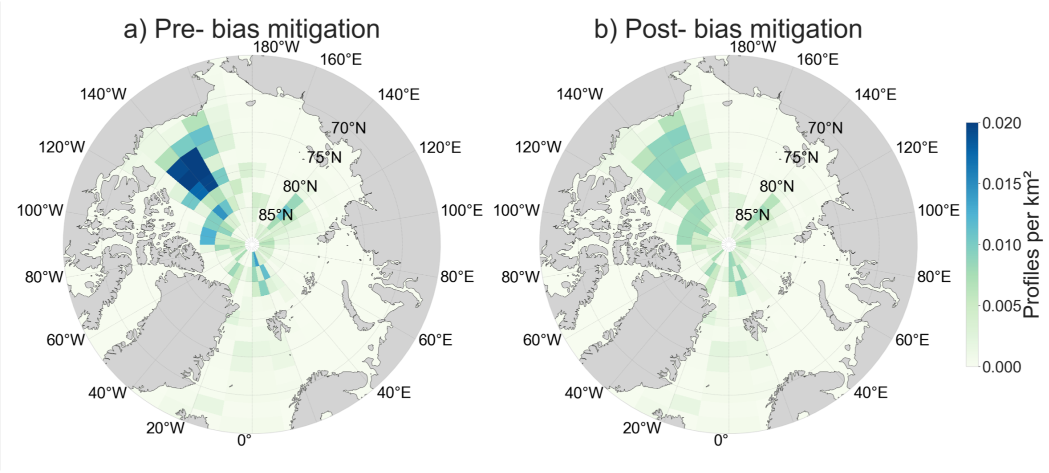

Figure 12: Spatial distribution of profile densities (profiles per km^2) in the Arctic Ocean before and after spatial and temporal bias mitigation for application of the Gaussian Mixture Model. Profile densities were obtained by normalising profile counts in 10° longitude bins and 2° latitude bins by the bin's surface area (in km^2, accounting for spherical geometry). Both maps use a common color scale (0–0.02 profiles per km^2) to enable direct comparison.

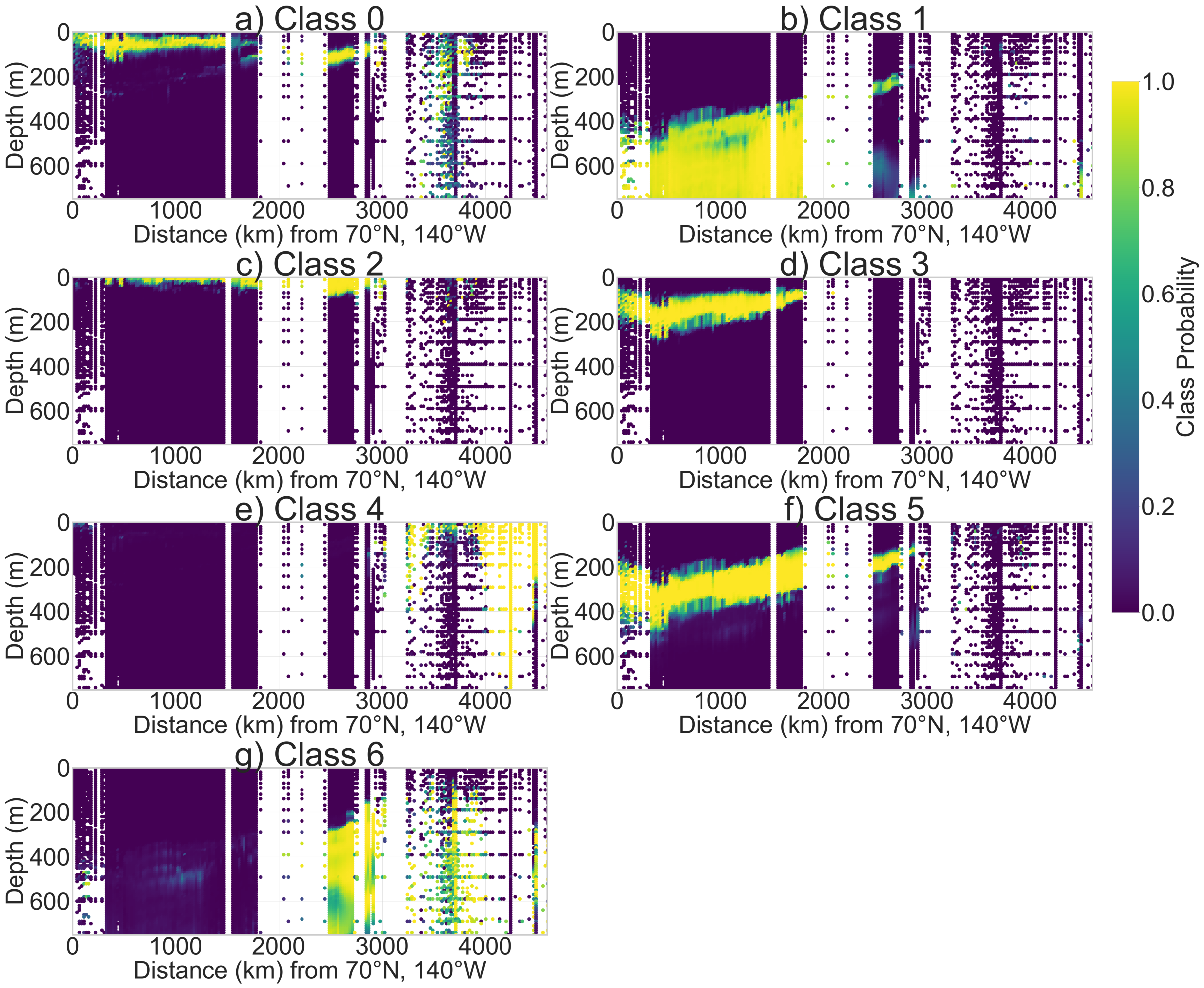

Figure 13: Latitudinal section of the probabilities generated by the GMM - representing the likelihood of each point to belong to each of the 7 classes: a-g) Classes 0-7. Section starts at the Canadian shelves at 70°N, 140°W (left) and ends at the Nordic Seas at 65°N, 0°E (right). The black solid line on the bathymetric map in Figure S6 marks the section location. Data are averaged into horizontal bins of 2.2km and vertical bins of 10m for visualisation purposes.

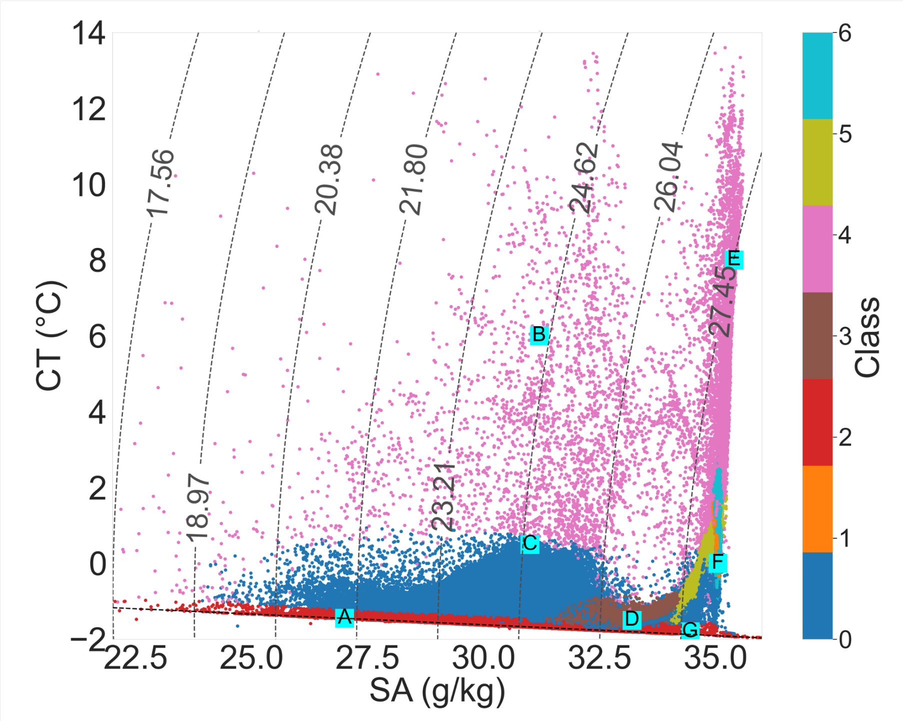

Figure 14: CT-SA diagram of all Arctic observational data compiled for this study, coloured by the dominant class (0-6) identified by the GMM. Observations without DO measurements are excluded. Cyan squares labels (A–G) denote the Source Water Types defined in our empirical study (see Table 2): A — ASW, B — sPW, C — MsPW, D — wPW, E — NCW), F — AW, and G — BW. Dashed curved contours mark potential density referenced to a pressure of 0 dbar (surface). The dashed horizontal line marks the freezing point of seawater.

References

[1] Peisong Zheng. Clustering under the ice. Msc dissertation, University of Cambridge, 2023. Supervised by Emma Boland.

[2] Donald J Cavalieri and Seelye Martin. The contribution of alaskan, siberian, and canadian coastal polynyas to the cold halocline layer of the arctic ocean. *Journal of Geophysical Research: Oceans*, 99(C9):18343–18362, 1994.

[3] Dorothea Bauch, Jens A Hölemann, Igor A Dmitrenko, Markus A Janout, Anna Nikulina, Sergey A Kirillov, Thomas Krumpen, Heidemarie Kassens, and Leo Timokhov. Impact of siberian coastal polynyas on shelf-derived arctic ocean halocline waters. *Journal of Geophysical Research: Oceans*, 117(C9), 2012.

[4] Ursula Schauer, Robin D Muench, Bert Rudels, and Leonid Timokhov. Impact of eastern arctic shelf waters on the nansen basin intermediate layers. *Journal of Geophysical Research: Oceans*, 102(C2):3371–3382, 1997.

[5] Leif G Anderson, Per S Andersson, Göran Björk, E Peter Jones, Sara Jutterström, and Iréne Wåhlström. Source and formation of the upper halocline of the arctic ocean. *Journal of Geophysical Research: Oceans*, 118(1):410–421, 2013.

[6] R Allyn Clarke, James H Swift, Joseph L Reid, and K Peter Koltermann. The formation of greenland sea deep water: double diffusion or deep convection? *Deep Sea Research Part A. Oceanographic Research Papers*, 37(9):1385–1424, 1990.

[7] Lynne D. Talley, George L. Pickard, and William J. Emery, editors. *Descriptive physical oceanography: an introduction*. Academic Press, Amsterdam ; Boston, 6th ed edition, 2011. OCLC: ocn720651296.

[8] John Marshall and Friedrich Schott. Open-ocean convection: Observations, theory, and models. *Reviews of geophysics*, 37(1):1–64, 1999.

[9] Graeme W. K. Moore, Kjetil Våge, Robert S. Pickart, and Ian A. Renfrew. Decreasing intensity of open-ocean convection in the greenland and iceland seas. *Nat. Clim. Change*, 5:877–882, 2015.

[10] Ursula Schauer. The release of brine-enriched shelf water from storfjord into the norwegian sea. *Journal of Geophysical Research: Oceans*, 100(C8):16015–16028, 1995.

[11] Detlef Quadfasel, Bert Rudels, and Stefan Selchow. The central bank vortex in the barents sea: watermass transformation and circulation. In *ICES MSS Vol. 195: Hydrobiological variability in the ICES Area, 1980–1989*, pages –. ICES, Copenhagen, Denmark, 1992.

[12] S. L. Pfirman, Dorothea Bauch, and T. Gammelsrød. The northern barents sea: Water mass distribution and modification. In *Proceedings of the AGU Geophysical Monograph Series*, pages –. American Geophysical Union, Washington, D.C., 1994.

[13] Frédéric Vivier, Antonio Lourenço, Ragnheid Skogseth, Ilona Goszczko, Elisabeth Michel, Clément Rousset, Pascale Bouruet-Aubertot, Yannis Cuypers, Bruno Lansard, and Claire Waelbroeck. Dense water production in storfjorden, svalbard, from a 1-year time series of observations and a simple model: Are polynyas in a warming arctic exporting heat to the deep ocean? *Journal of Geophysical Research: Oceans*, 129(10):e2024JC020878, 2024.

[14] Ragnheid Skogseth, Peter M. Haugan, and Martin Jakobsson. Watermass transformations in storfjorden. *Continental Shelf Research*, 25(5-6):667–695, 2005.

[15] Axel Behrendt, Hiroshi Sumata, Benjamin Rabe, and Ursula Schauer. Udash – unified database for arctic and subarctic hydrography [dataset]. *Earth Syst. Sci. Data*, 10(2):1119–1138, June 2018.

[16] John M. Toole, Richard A. Krishfield, Mary Louise Timmermans, and Andrey Proshutinsky. The Ice-Tethered profiler: ArgoEuroArgoDataSelection of the Arctic. *Oceanography*, 24(3), 2011.

[17] Siv K. Lauvset, Nico Lange, Toste Tanhua, Henry C. Bittig, Are Olsen, Alex Kozyr, Simone Alin, Marta Álvarez, Kumiko Azetsu-Scott, Leticia Barbero, Susan Becker, Peter J. Brown, Brendan R. Carter, Leticia Cotrim da Cunha, Richard A. Feely, Mario Hoppema, Matthew P. Humphreys, Masao Ishii, Emil Jeansson, Li-Qing Jiang, Steve D. Jones, Claire Lo Monaco, Akihiko Murata, Jens Daniel Müller, Fiz F. Pérez, Benjamin Pfeil, Carsten Schirnick, Reiner Steinfeldt, Toru Suzuki, Bronte Tilbrook, Adam Ulfsbo, Anton Velo, Ryan J. Woosley, and Robert M. Key. Glodapv2.2022: the latest version of the global interior ocean biogeochemical data product [dataset]. *Earth System Science Data*, 14(12):5543–5572, 2022.
